# Tricolor visible wavelength-selective photodegradable hydrogel biomaterials

**DOI:** 10.1038/s41467-023-40805-w

**Published:** 2023-08-29

**Authors:** Teresa L. Rapp, Cole A. DeForest

**Affiliations:** 1 Department of Chemical Engineering, University of Washington, Seattle, WA 98195 USA; 2 Department of Bioengineering, University of Washington, Seattle, WA 98195 USA; 3 Department of Chemistry, University of Washington, Seattle, WA 98195 USA; 4 Institute of Stem Cell & Regenerative Medicine, University of Washington, Seattle, WA 98109 USA; 5 Molecular Engineering & Sciences Institute, University of Washington, Seattle, WA 98195 USA; 6 Institute for Protein Design, University of Washington, Seattle, WA 98195 USA

**Keywords:** Biomaterials, Photochemistry, Biomaterials, Soft materials

## Abstract

Photodynamic hydrogel biomaterials have demonstrated great potential for user-triggered therapeutic release, patterned organoid development, and four-dimensional control over advanced cell fates in vitro. Current photosensitive materials are constrained by their reliance on high-energy ultraviolet light (<400 nm) that offers poor tissue penetrance and limits access to the broader visible spectrum. Here, we report a family of three photolabile material crosslinkers that respond rapidly and with unique tricolor wavelength-selectivity to low-energy visible light (400–617 nm). We show that when mixed with multifunctional poly(ethylene glycol) macromolecular precursors, ruthenium polypyridyl- and *ortho*-nitrobenzyl (*o*NB)-based crosslinkers yield cytocompatible biomaterials that can undergo spatiotemporally patterned, uniform bulk softening, and multiplexed degradation several centimeters deep through complex tissue. We demonstrate that encapsulated living cells within these photoresponsive gels show high viability and can be successfully recovered from the hydrogels following photodegradation. Moving forward, we anticipate that these advanced material platforms will enable new studies in 3D mechanobiology, controlled drug delivery, and next-generation tissue engineering applications.

## Abstract (web summary)

Photodegradable gels are useful for regenerative medicine and tissue engineering, but typically respond to UV light. Here, the authors report a family of 3 photolabile linkers that cleave rapidly and with unique tricolor wavelength-selectivity to visible light.

## Introduction

Light is a particularly powerful stimulus for gaining spatiotemporal control over dynamic material transformations. In contrast to intrinsic stimuli such as pH, temperature, and protease levels that are challenging to regulate in a biological setting, light’s ability to be precisely administered affords unique control over when, where, and to what degree materials undergo pre-programmed responses as well as a route to sidestep challenges associated with patient-to-patient variability in clinical applications. Recognizing this unique utility, the biomaterials community has invested substantial efforts towards the development of photoresponsive hydrogels – water-swollen polymer networks that elicit a prespecified change upon directed light irradiation^1–4^. The resulting systems have driven significant advances across multiple fields, including cellular biology^5–10^, drug discovery^11–15^, tissue engineering platforms for directing cell fate^16,17^, and organoid culture^18,19^. Photodynamic hydrogels take advantage of light’s spatiotemporal control to trigger photoinduced material softening^20,21^, biochemical release^22–24^, and species uncaging/activation for in vivo material modification^25^.

Despite the early promise of phototunable gels, chemistry- and material-based limitations have precluded the full power of these platforms from being realized^2^. Reliance on organic chromophores to encode photodegradability within hydrogels has historically confined the photocleaving wavelengths to high-energy light–ranging from ≤365 nm for *ortho*-nitrobenzyl (*o*NB)-based systems up to 450 nm for some reported photolabile coumarins and bimanes^26–28^. While some of these chromophores have been highly characterized and are cytocompatible throughout photolysis, the utilization of UV light irradiation has traditionally confined these materials to solely in vitro use as high-energy light is poorly penetrant through complex tissue^3,29^. Additionally, only a limited number of reports have described materials that are sensitive to multiple orthogonal wavelengths, of which nearly all crosslinkers employed rely heavily on near- and far-UV light^27,28,30–33^, thereby missing significant opportunities for cytocompatible material multiplexing by taking advantage of the entire spectrum of visible and IR light. A collection of photocleavable crosslinkers that distinctly responds to an expanded portion of electromagnetic spectrum, enabling deeper tissue penetration and multiplexed wavelength-selective material modulation using differently colored light, would enable exciting new directions for phototunable hydrogel platforms.

Seeking to expand light response into the visible range, we turned our attention towards ruthenium (Ru) polypyridyl complexes, which continue to gain new and unique ground in the world of photochemistry^34,35^. These compounds are highly photoactive and undergo covalent bond breakage upon visible light irradiation, with quantum yields of photodissociation many times greater than current organic chromophores and reaching 0.1–0.6 in many cases^30,36,37^. Capitalizing on these advances, Ru complexes have found utility in the biochemical space as photocaging groups^35,38,39^ and in the generation of photoresponsive micelles for drug delivery^40^. Though most ruthenium compounds bearing 2,2’-bipyridine ligands commonly absorb blue light (420–450 nm), comparatively simple syntheses can yield species with shifted absorbances extending into the red and near-IR^41,42^. With their radical-free ligand exchange^39^, engineered cytocompatibility, and high photoefficiency, we postulated that uniquely visible light-responsive biomaterials could be created through direct inclusion of such Ru complexes in a polymer backbone, providing a route to next-generation photodynamic hydrogels.

**Fig. 1:**
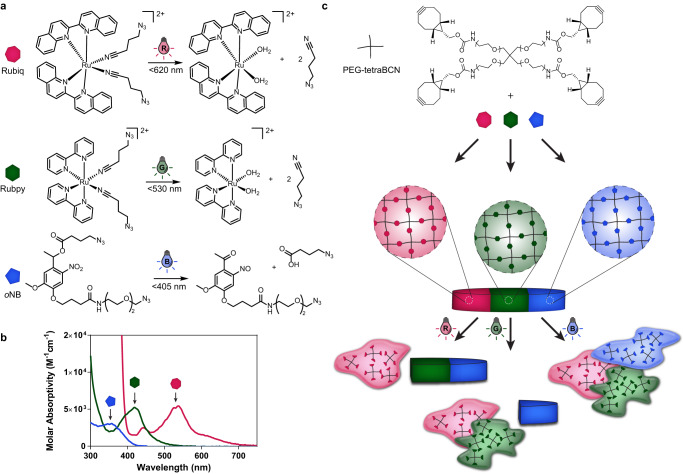
Photodegradable crosslinkers cleave in a visible wavelength-selective manner. **a** Novel crosslinkers Rubiq (pink heptagon), Rubpy (green hexagon), and *o*NB (blue pentagon) undergo photolysis. Upon absorption of a photon, each ruthenium complex undergoes ligand exchange via population of a triplet metal-centered state via intersystem crossing from the low-lying metal-to-ligand charge transfer (MLCT) band, resulting in heterolytic ligand/solvent exchange. Crosslinkers are modified with two azides for direct incorporation into hydrogel networks. **b** Absorbance spectra of Rubiq, Rubpy, and *o*NB. Each crosslinker exhibits an extended absorbance spectrum beyond their λ_max_ permitting excitation at wavelengths lower than λ_max_. **c** PEG-based hydrogel formation occurs spontaneously upon mixing each crosslinker with 4-arm PEG-BCN via strain-promoted azide-alkyne cycloaddition. Resulting hydrogels degrade under red, green, or blue light with perfect unidirectional orthogonality (>617 nm for Rubiq, >530 nm for Rubpy, and >405 nm for *o*NB).

In this work, we describe the synthesis and properties of a unique series of photolabile crosslinkers, including two based on ruthenium compounds: (1) Ru(2,2’-biquinoline)_2_(4-azidobutanenitrile)_2_ (denoted Rubiq); (2) (Ru(2,2’-bipyridine)_2_(4-azidobutanenitrile)_2_ (Rubpy); and (3) an established *o*NB^43^ with wavelength-separated λ_max_ capable of respectively responding selectively to three different colors of visible light – red (617 nm), green (530 nm), and blue (405 nm) (Fig. 1a, b). Mixing four-arm poly(ethylene glycol) (PEG) end-functionalized with bicyclononyne (PEG-tetraBCN) together with the bis(azide)-modified photolinkers with 1:1 stoichiometry with respect to their reactive groups, idealized step-growth polymeric gels can be readily formed through bioorthogonal strain-promoted azide-alkyne cycloaddition (SPAAC) (Fig. 1c)^44^. Taking advantage of crosslinkers designed to rapidly photolyze in a cytocompatible and visible wavelength-selective manner, we anticipated being able to achieve multiplexed material degradation in the presence of living cells and through complex tissue.

## Results

### Synthesis and photochemical characterization of Rubiq, Rubpy, and *o*NB-based crosslinkers

Photolabile crosslinkers were each synthesized with flanking azides, permitting their incorporation into bulk hydrogels via SPAAC. Rubiq and Rubpy were respectively synthesized in a two- and one-step processes from Ru(bpy)_2_Cl_2_ or RuCl_3_ and 2,2’-biquinoline, whereby azide-bearing ligands (4-azidobutanenitrile) were coordinated to form symmetrical Ru-based crosslinkers. The bis(azide) *o*NB was synthesized following published protocol with minimal changes^43^ (see Methods for synthetic details). All crosslinkers were synthesized, purified, and characterized by ^1^H NMR and electrospray ionization mass spectroscopy (ESI-MS, Supplementary Method 1, Supplementary Figs. 1–6) with appreciable overall yields (Rubiq, 27%; Rubpy, 77%; *o*NB, 17%).

**Fig. 2:**
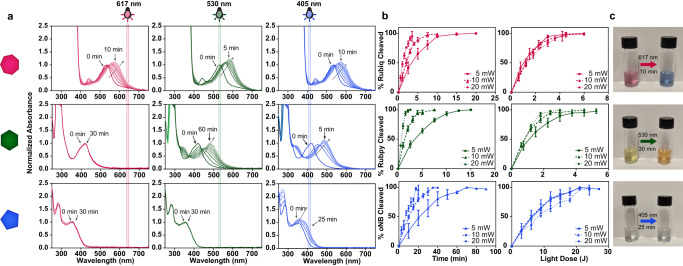
Photolabile crosslinkers can be selectively cleaved with differently colored visible light. **a** Rubiq (pink heptagon), Rubpy (green hexagon), and *o*NB (blue pentagon) individually subjected to 617, 530, and 405 nm light (10 mW cm^−2^, 0–60 min) undergo wavelength-selective photolysis, indicated by absorbance spectral changes. **b** Photolysis proceeds efficiently with varied power (5, 10, 20 mW) in a light dose-dependent manner. Here, light dosage is calculated as the product of light intensity and exposure time. *n* = 3 for each photolysis, error bars represent standard deviation about the experimental mean. **c** Color change was observed for Rubiq and Rubpy upon irradiation (10 mW, varied wavelengths and exposure times) due to the significant absorbance shift following ligand exchange.

Upon irradiation with the appropriate wavelengths of light, Rubiq and Rubpy undergo ligand exchange with a solvent molecule (in this case, water) due to population of a metal-centered antibonding orbital (Fig. 1a, Supplementary Fig. 7). Rubiq and Rubpy exhibit low-energy absorbance bands due to low-lying metal-to-ligand charge transfer (MLCT) states common in ruthenium polypyridyl complexes^45^. As expected, the extended pi bond structure of 2,2’-biquinoline gave a bathochromic shift in peak absorbance of Rubiq to a λ_max_ of 535 nm, significantly red shifted when compared with the 2,2’-bipyridine-containing Rubpy compound and *o*NB^46^. All crosslinkers exhibited a significant λ_max_ with a slow decay in absorptivity extending well into the visible range, permitting off-peak photon absorbance and population of antibonding orbitals at low energies (Fig. 2a). For this study, Rubiq was photocleaved using 617 nm (ε = 43 M^−^^1^cm^−^^1^), Rubpy with 530 nm (ε = 14 M^−^^1^cm^−^^1^), and *o*NB at 405 nm (ε = 118 M^−^^1^cm^−^^1^), wavelengths commonly accessible through inexpensive LED light sources that balanced photocleavage efficiency and spectral separation.

To probe the mechanism of photomediated ligand exchange in the Ru crosslinkers, Rubiq and Rubpy (5 mM) were analysed by ^1^H NMR and ESI-MS over 60 min of exposure (10 mW cm^−^^2^, 617 nm and 7.8 mW cm^−^^2^, 530 nm respectively). ^1^H NMR revealed exchange of both azide-bearing ligands within the first 2.5 minutes of light exposure, Supplementary Figs. 8, 9). This trend was further confirmed through ESI-MS analysis, which additionally indicated that 2,2’-biquinoline and 2,2’-bipyridine ligands remain attached to Ru as expected (Supplementary Figs. 10, 11). Motivated by an interest to use these in the presence of living cells, we further analysed whether singlet oxygen (^1^O_2_) generation would accompany crosslinker photolysis, as it does when similar compound Ru(bpy)_3_ is exposed to light^47,48^. While ^1^O_2_ was detected for all linkers, each yielded statistically significantly less ^1^O_2_ than Ru(bpy)_3_ under matched illumination conditions, with Rubpy producing less than Rubiq and *o*NB (Supplementary Fig. 12). Since *ortho*-nitrobenzyl-based crosslinkers have been extensively demonstrated as cytocompatible and Ru(bpy)_3_ is regularly exploited as a visible light-mediated photoinitiator for cell encapsulation, these results were encouraging.

This series of crosslinkers was shown to be unidirectionally orthogonal: red light (617 nm) irradiation led to photolysis of Rubiq only, green light (530 nm) irradiation photolyzed both Rubiq and Rubpy but left *o*NB intact, and blue light (405 nm) photolyzed all three crosslinkers (Fig. 2a). This is demonstrated by the presence or absence of a shift in absorbance spectrum upon irradiation; all three compounds undergo a change in their electronic structure upon photolysis which is reflected in the absorbance spectra. The insensitivity of Rubpy to high doses of red light, as well as *o*NB to extended exposure to red and green light, demonstrates wavelength selectivity of the small molecule crosslinkers in situ.

The appearance of a new absorbance maximum of photoproducts was used to determine the quantum yield (QY) of photocleavage at the wavelengths used in this study (Fig. 2b, Table 1). Light at 617 nm, 530 nm, and 405 nm at 5, 10, and 20 mW were used for the QY determination. As expected, faster photocleavage for each compound accompanied higher light intensities. When extent of photolysis was normalized to total light dosage (i.e., the product of intensity and exposure time), cleavage traces collapsed onto a single curve for each species (Fig. 2b). Rubpy has the highest QY of the three (0.29 ± 0.04), consistent with prior reports for Ru(bpy)_2_L_2_ complexes under visible light irradiation^30,37,49^. Rubiq’s QY (0.016 ± 0.009) was found to be lower than that of Rubpy, due to the bathochromic shift decreasing intersystem crossover efficiency into the metal-centered antibonding orbital^46^. Though *o*NB has a very low QY at 405 nm (0.004 ± 0.003), its higher molar absorptivity enables photocleavage under visible irradiation with reasonable efficiency. Photolysis was found to be complete for all three crosslinkers within 30 minutes at 10 mW (Fig. 2b). Significant color change accompanied photolysis for each compound, particularly for Rubiq, providing a useful handle for qualitative crosslinker cleavage determination in hydrogel formulations (Fig. 2c).

**Table 1:** Photokinetic constants

Compound	λ_max_ (nm)	λ_excitation_ (nm)	Quantum Yield (Φ_pr_)	Molar Absorptivity (ε, M^−^^1^cm^−^^1^)	Efficiency (Φ • ε)
Rubiq	535	617	0.016 ± 0.009	43	0.688
Rubpy	420	530	0.29 ± 0.04	14	4.06
*o*NB	365	405	0.004 ± 0.003	118	0.472

### Multiplexed hydrogel degradation via visible light irradiation

**Fig. 3:**
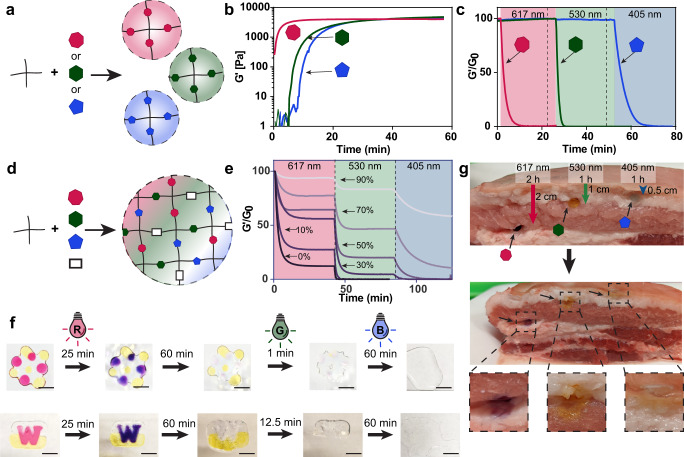
Wavelength-orthogonal hydrogel degradation via red, green, and blue light. **a** Hydrogels formed via SPAAC chemistry between PEG-tetraBCN and the azide-flanked crosslinkers. **b** Rubiq (pink heptagon)-, Rubpy (green hexagon)-, and *o*NB (blue pentagon)-crosslinked hydrogels form equivalently stiff hydrogels (G’ ~ 2-3 kPa) as determined rheometrically. **c** Individually crosslinked hydrogels are unidirectionally orthogonally degraded, with stable storage modulus observed in Rubpy-crosslinked gels under red light irradiation and *o*NB-crosslinked gels under red and green light irradiation. **d** Hydrogels were formed with equal ratios of all three photolabile crosslinkers and increasing amounts of nondegradable diazido-tri(ethylene glycol) (TEG) crosslinker (0–90%, white rectangle). **e** Intermediate stiffnesses were accessible through partial degradation of hydrogel crosslinks. Hydrogels were exposed to red, green, then blue light (35 min each, 10 mW cm^−^^2^) to selectively cleave specific amounts of crosslinks within the material. Reported percentages refer to the amount of stable, non-cleavable crosslink present in the material. **f** Spatial control over hydrogel degradation demonstrated by the casting of individually crosslinked hydrogels in close proximity. Open microfluidic methodologies were used to cast interconnected multimaterial geometries; exposure to red light resulted in the center Rubiq degradation, observed after a 60- min wash. Subsequent exposures to green and blue light rapidly degraded the Rubpy and *o*NB portions, respectively. Scale bar = 5 mm. **g** Rubiq hydrogels were completely degraded through up to 2 cm of complex tissue (skin-on pork belly shown here), Rubpy hydrogels through 1 cm, and *o*NB (dyed with Cy5) through 0.5 cm.

Having demonstrated the wavelength orthogonality and high efficiency of this series of photocleavable molecules, we sought to employ them as crosslinkers in PEG-based hydrogel systems. To create visible light-degradable hydrogels, Rubiq, Rubpy, and *o*NB were polymerized with PEG-tetraBCN via SPAAC chemistry (Fig. 3a). Though gelation kinetics varied slightly with crosslinker identity, each formulation resulted in equivalently stiff hydrogels (G’ ~ 2.5 kPa), as quantified using in situ rheometry (Fig. 3b). Following complete gelation, hydrogels crosslinked with Rubiq, Rubpy, and *o*NB were individually subjected to sequential treatments (35 min each) of 617 nm, 530 nm, and 405 nm light (10 mW cm^−^^2^), with photodegradation assessed rheometrically. Rubiq-based gels degraded rapidly upon red-light illumination, while those containing Rubpy and *o*NB persisted; Rubpy subsequently degraded with green light with *o*NB materials unchanged; finally, blue light afforded rapid cleavage of the remaining *o*NB-crosslinked hydrogel (Fig. 3c). Hydrogel stability was dependent on temperature: when stored in the dark, Rubiq-crosslinked hydrogels were stable (defined as <30% degradation, wherein hydrogels swelled but maintained their original shape and structural integrity) at room temperature for 24 h, but were fully dissolved by this timepoint when stored at 37 °C; Rubpy hydrogels were stable for 7 days at room temperature, but only 48 h at 37 °C; *o*NB-crosslinked hydrogels are considered completely stable, experiencing no loss in material integrity over the course of the experiment at both temperatures (Supplementary Fig. 13). Results highlight that the gels can be photodegraded in a visible wavelength-selective manner as designed.

To access intermediate bulk hydrogel stiffnesses, hydrogels were cast with equal amounts of the photolabile crosslinkers (i.e., 1:1:1 Rubiq:Rubpy:*o*NB) alongside varied percentages (0–90%) of photostable diazido-tri(ethylene glycol) crosslinker (denoted TEG), while keeping the total crosslinker concentration constant (Fig. 3d, Supplementary Fig. 14). When sequentially subjected to red, green, and blue light (10 mW cm^−^^2^, 35 min each) and analyzed rheometrically, hydrogels partially softened to intermediate moduli or fully degraded in a manner dependent on crosslinker makeup and wavelength-selectivity. Consistent with polymer network rubber elasticity theory, the incorporation of stable TEG crosslinks into the matrix at varying percentages stabilized the hydrogel at intermediate stiffness following complete lysis of photocleavable crosslinks. Hydrogels with low percentages of TEG (10%) lost all elastic properties after exposure to red+green light. When TEG comprised >60% of crosslinks, the hydrogel was no longer fully photodegradable, yielding a soft stable hydrogel following complete light treatment (Fig. 3e).

To illustrate that wavelength-selectivity could be used to dynamically pattern hydrogel multimaterials, we formulated multilayered hydrogels composed of distinctly patterned regions of Rubiq, Rubpy, and *o*NB using open-microfluidic additive manufacturing methods^50^. These multifunctional hydrogels were sequentially exposed to red, green, and blue light, and imaged using digital photography (Fig. 3f). Following red-light illumination, Rubiq exhibited the pink-to-purple color change observed previously to accompany degradation, while Rubpy and *o*NB were visibly unaltered. Treatment with green light caused Rubpy to lose its yellow color and degrade, leaving only *o*NB regions intact. Final treatment with blue light yielded complete material dissolution.

Encouraged by the hydrogel’s rapid response to low-energy visible light, we next sought to examine whether hydrogels could be degraded through complex tissue. Skin-on pork belly was dissected and hydrogels placed at varying depths; a Rubiq-crosslinked hydrogel was deposited 2 cm below the skin surface, a Rubpy hydrogel at 1 cm, and *o*NB (modified with Cy5-azide to enhance visualization) at 0.5 cm. Gels were respectively subjected to red, green, and blue light (50 mW cm^−^^2^ at the skin) and in-tissue degradation was visually monitored (Fig. 3h). Hydrogel dissolution was observed for all three hydrogels following <2 h illumination at the depths reported, mirroring previously reported studies with Ru-based photocages^51^. To the best of our knowledge, this is the first time that a photodegradable biomaterial has been directly controlled at these tissue depths, highlighting the potential of these materials for future in vivo applications.

### Cell viability throughout encapsulation and release from tricolor-responsive hydrogels

Having demonstrated that hydrogel responses could be controlled in a wavelength-specific manner using visible light, we next sought to investigate the cytocompatibility of these crosslinkers in solution, as well as during gel formation and subsequent photodegradation. Human bone marrow-derived stromal cells (hS5s) and mouse 10T1/2 immortalized fibroblasts were exposed to Rubiq and Rubpy (1 mM) for 3 h prior to proliferation assessment via longitudinal viability assay (RealTime Glo, Promega, Supplementary Figs. 15, 16). Rubiq exposure led to significant loss in viability as well as an arrest of the cell cycle for both cell types. Rubpy exhibited no toxicity for hS5s, and only modest decrease in early 10T1/2 proliferation rates. To probe the mechanism of cell death, we measured caspase 3 activity levels as a measure of apoptosis 24 h following crosslinker exposure and found no significant differences, suggesting an alternative mechanism (Supplementary Figs. 17, 18). Interestingly, pre-reacting both Rubiq and Rubpy with a stoichiometric excess of monofunctionalized methoxy-PEG-BCN yielded di-PEGylated species that was markedly less toxic than the free crosslinkers. These results suggest that crosslinker toxicity is greatly reduced upon polymerization into a PEG-based hydrogel network (Supplementary Figs. 15, 16).

**Fig. 4:**
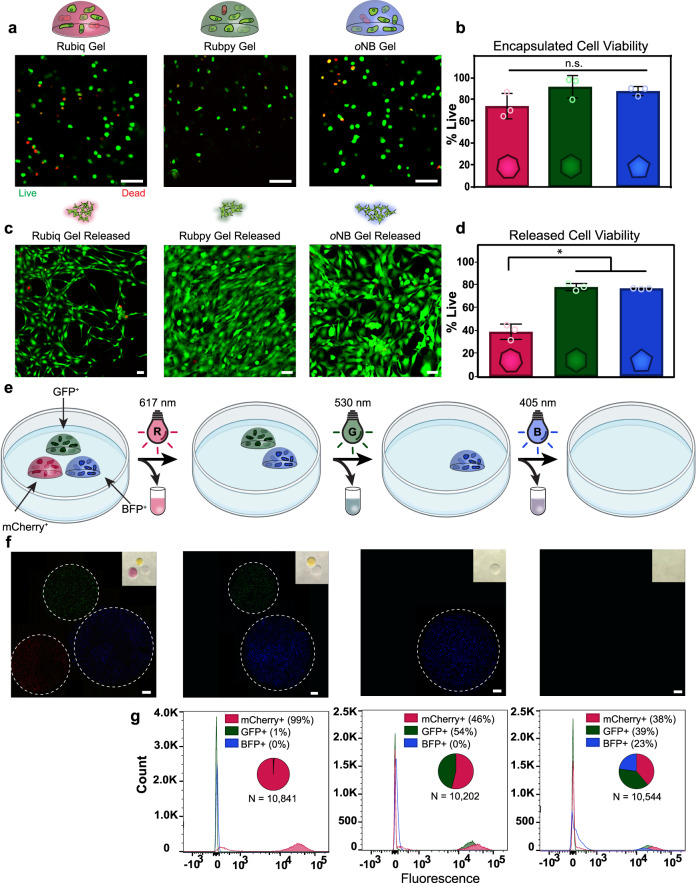
Cell viability following encapsulation in and photorelease from Rubiq, Rubpy, and *o*NB-crosslinked hydrogels. **a** Live/dead staining of 10T1/2 fibroblasts encapsulated in individually crosslinked hydrogels, shown as single z-slice obtained via confocal micrography. Calcein (green) and ethidium homodimer (EtHD, red) dyes were used for live and dead staining respectively. Scale bar = 50 μm. **b** Encapsulated cell viability at 24 h post encapsulation (cell counts collected from 40 μm x 1 mm × 1 mm section through the vertical center of the hydrogel). Cell counting revealed no statistical difference (**p** > 0.05, one-way ANOVA) in cell viability across all crosslinkers at 24 h. *n* = 3 individual biological replicates, error bars represent standard deviation about the experimental mean. **c** Rubiq, Rubpy, and *o*NB hydrogels were exposed to red, green, or blue light respectively (617 nm, 530 nm, 405 nm; 10 mW cm^−^^2^) and the released fibroblasts allowed to settle to the bottom of the well over 24 h prior to imaging. Surviving cells exhibited classical fibroblast morphology upon calcein green/EtHD staining. Scale bars = 50 μm. **d** High levels of viable cells were recovered from each hydrogel formulation, though cells released from Rubiq hydrogels showed lower levels of viability compared to Rubpy and *o*NB. ^**^p = 0.0036 by one-way ANOVA, *n* = 3 individual biological replicates, error bars represent standard deviation about the experimental mean. **e** mCherry^+^, GFP^+,^ and BFP^+^ hS5 cells were encapsulated in Rubiq, Rubpy, and *o*NB hydrogels respectively. 24 h after encapsulation, hydrogels were exposed to 617 nm, 530 nm, or 405 nm light. **f** Images of hydrogels bearing mCherry^+^, GFP^+^, or BFP^+^ hS5 cells following light exposure. Scale bars = 500 μm. Experiment performed in biological triplicate with similar results; representative images given. **g** Flow cytometry histograms show only expected cell populations were released into the media, demonstrating the orthogonality of the hydrogel system.

To determine cell survival throughout encapsulation in a hydrogel, hS5s, human mesenchymal stem cells (hMSCs), and 10T1/2 cells were encapsulated (5 × 10^6^ cells mL^−^^1^) in Rubiq, Rubpy, and *o*NB-crosslinked hydrogels (Fig. 4a, Supplementary Figs. 19, 20). Live/dead staining with calcein/ethidium homodimer and fluorescent confocal imaging was performed 24 h after encapsulation. Reasonably high viability (between 40–95%) was observed in all cases (Fig. 4b, c), consistent with SPAAC’s reported biocompatibility for gel formation and that of the crosslinkers^52^, though some cell- and crosslinker-type dependencies were observed.

Photorelease from hydrogels had a significant effect on viability, with results again varying across the three different cell types. To assess the cytocompatibility of photodegradation, cell-laden hydrogels were photodegraded with red, green, or blue visible light 1 day post encapsulation. In a subset of the gels, released cells were left suspended in hydrogel photoproducts for an additional 24 h, after which the cells that adhered were stained for viability (Fig. 4c). Fibroblasts showed normal morphology 24 h after plating, suggesting the photodegradation process did not alter them significantly. To confirm viability following release, cells were collected following hydrogel degradation and assessed directly for viability via flow cytometry (Fig. 4d, Supplementary Fig. 21). Though viability was generally high, results demonstrated that fibroblasts released from Rubiq-crosslinked gels exhibited a significant loss in viability of approximately 40%, suggesting that the Rubiq photoproduct may be partially cytotoxic. Proliferation rates of gel-released hS5s and hMSCs were also assessed via EdU (5-ethynyl-2´-deoxyuridine) staining and quantification of newly synthesized DNA; while hMSC cells showed minimal EdU^+^ nuclei, hS5s showed significant recovery of proliferative behaviour following photorelease, but again with poorer performance in the Rubiq-crosslinked systems (Supplementary Figs. 19, 20). Prior studies on this type of compound suggests the extended aromatic ligands on Ru compounds contribute to their instability and enhanced cytotoxicity^53^, though future designs may include incorporation of hydrophilic groups to decrease cellular uptake leading to their toxicity^42^.

Taking advantage of the spatial resolution afforded by this hydrogel system using orthogonal wavelengths of light, unique cell populations were encapsulated and recovered from individual hydrogels. hS5s stably expressing either mCherry, GFP, or BFP were encapsulated in Rubiq, Rubpy, and *o*NB-crosslinked hydrogels, respectively (Fig. 4e). Upon irradiation with red, green, or blue light, selective hydrogel degradation was observed by confocal microscopy (Fig. 4f). Following degradation, the gel-liberated cell populations were collected and analyzed via flow cytometry. Harvested cell collections matched the expected color composition: 617 nm light released only mCherry^+^ cells, 530 nm yielded matched populations of mCherry^+^ and GFP^+^, and 405 nm light degraded all three hydrogels and gave equivalent counts of mCherry^+^, GFP^+^, and BFP^+^ cells (Fig. 4g, Supplementary Fig. 22). Results further demonstrate wavelength orthogonality and multiplexability of cell release from tricolor visible light-responsive gels.

## Discussion

In summary, photolabile crosslinkers Rubiq, Rubpy, and *o*NB provide a route towards the first tricolor visible light-degradable gel system. We have established that these crosslinkers are synthetically accessible with moderate yields and photoresponsive to low-energy light. These species are highly photoactive under visible light irradiation permit facile synthesis of PEG-based hydrogels that rapidly and orthogonally degrade under red (617 nm), green (530 nm) and blue (405 nm) light. The use of low-energy light permits hydrogel degradation through complex tissue up to 2 cm in depth, while wavelength-orthogonality enables sequential and spatial control over hydrogel degradation. We have demonstrated crosslinker cytocompatibility, with moderate-to-high viability observed for three distinct cell types upon hydrogel encapsulation and 3D culture. Exploiting their wavelength-selective degradation, we demonstrated multiplexed cell populations recovery, a feature that is likely to be useful for in vivo cell delivery applications. Moving forward, we anticipate that these materials will prove enabling for additional applications in mechanobiology, 4D cell culture, and controlled therapeutic release.

## Methods

### General synthetic methods

All reactions and purification protocols were performed under red light. All solvents and chemicals were used as received with no further purification. All reagents and solvents were obtained from Fisher Scientific unless otherwise stated and used without further purification.

#### 4-azidobutyronitrile

4-bromobutyronitrile (1 mL, 10 mmol) and sodium azide (1.3 g, 20 mmol) were dissolved in dimethylsulfoxide (DMSO, 15 mL) and stirred overnight at 55 °C. The reaction was cooled to room temperature, diluted with water to 50 mL, extracted with diethyl ether (3 × 50 mL), dried over Na_2_SO_4_, and the ether was removed under vacuum to give the product as a light-yellow oil. SAFTEY HAZARD: 4-azidobutyronitrile is isolated with DMSO in the final form. It should never be fully dried, as it is an explosive small molecule azide when concentrated. Yield: 1.017 g, 92%. ^1^H NMR: (CDCl_3_, 300 MHz) δ 3.50 (t, 2H, *J* = 6.34), 2.48 (t, 2H, *J* = 7.05), 1.92 (quint, 2H, *J* = 12.96).

#### Ru(biquinoline)_2_Cl_2_ (Ru(biq)_2_Cl_2_)

RuCl_3_ (anhydrous, 99.96% trace metal basis, 400 mg, 1.93 mmol), hydroquinone (444 mg, 4 mmol), and LiCl (480 mg, 11.3 mmol) were suspended in dimethylformamide (DMF, 10 mL) and bubbled with N_2_ for 15 min. 2,2’-biquinoline (1 g, 4 mmol) was added, and the reaction was heated to 130 °C for 1 h until color appeared forest green, then cooled to room temperature. Reaction was added dropwise to 800 mL deionized water and filtered to collect a dark green-black solid. Solid was dissolved in dichloromethane (200 mL), solvent was reduced to 100 mL under vacuum, then added to diethyl ether (400 mL) and filtered to collect Ru(biq)_2_Cl_2_ as a dark green solid. Product was used without further purification. Yield: 805 mg, 61%.

#### Ru(biq)_2_(4-azidobutyronitrile)_2_[Cl_2_] (**Rubiq**)

Ru(biq)_2_Cl_2_ (250 mg, 0.365 mmol) and silver hexafluorophosphate (275 mg, 1.08 mmol) were dissolved in methanol (40 mL, stored over molecular sieves) and stirred at 55 °C in the dark until reaction turned blue and white silver chloride powder was observed (15 min). 4-azidobutyronitrile (100 µL, 0.945 mmol) was added, and the reaction stirred at 55 °C for 1 h until color turned pink. Reaction was cooled to room temperature, filtered to remove silver chloride, and methanol was removed by rotary evaporation. Ru(biq)_2_(4-azidobutyronitrile)_2_ was purified by silica flash chromatography with 1:4 acetonitrile:dichloromethane as the eluent.

Ru(biq)_2_(4-azidobutyronitrile)_2_[PF_6_]_2_ was converted to the chloride salt by anion-exchange resin. RuPink[PF_6_]_2_ was passed over Amberlite IRA-410 (Cl Form) resin using methanol as the eluent, giving Ru(biq)_2_(4-azidobutyronitrile)_2_[Cl]_2_ as the water-soluble form (denoted﻿ Rubiq). Yield: 159 mg, 48%. Overall yield: 27% ^1^H NMR: (CD_3_CN, 300 MHz) δ 9.14 (broad s, 2H), 8.68 (d, 2H, *J* = 8.69), 8.36 (d, 2H, *J* = 7.99), 8.31 (d, 2H, *J* = 8.09), 8.21 (broad s, 2H), 8.02 (t, 2H, *J* = 7.27), 7.91 (d, 2H, *J* = 7.84), 7.50 (t, 2H, *J* = 7.04), 6.87 (t, 2H, *J* = 7.17), 6.75 (d, 2H, *J* = 8.4), 3.08 (t, 4H, *J* = 6.42), 2.75 (t, 4H, *J* = 7.25), 1.65 (quint, 4H, *J* = 6.57). Expected mass [H^+^, m/2]: 417.11, observed mass: 417.1125 (m/2).

#### Ru(bpy)_2_(4-azidobutyronitrile)_2_[Cl_2_]  (**Rubpy**)

Ru(bipyridine)_2_Cl_2_•2H_2_O (200 mg, 0.38 mmol) and silver hexafluorophosphate (212 mg, 0.83 mmol) were dissolved in methanol (stored over molecular sieves, 40 mL) and stirred at 55 °C for 15 min. 4-azidobutyronitrile (390 µL, 3.7 mmol) was added and the reaction stirred at 55 °C for 2 h until color changed from red to orange. Reaction mixture was cooled to room temperature and filtered to remove silver chloride, then concentrated by rotary evaporation. The product (denoted Rubpy) was purified by silica flash chromatography with 4:1 dichloromethane:acetonitrile as the eluent, collecting the major yellow band. Yield: 22 mg, 84%. Overall yield: 77% ^1^H NMR: (CD_3_CN, 300 MHz) δ 9.61 (d, 2H, *J* = 4.95), 8.83 (d, 2H, *J* = 8.07), 8.69 (d, 2H, *J* = 8.10), 8.43 (t, 2H, *J* = 7.16), 8.12 (t, 2H, *J* = 7.16), 8.00 (t, 2H, *J* = 6.00), 7.93 (d, 2H, *J* = 5.70), 7.46 (t, 2H, *J* = 6.00), 3.37 (t, 4H, *J* = 6.33), 3.00 (t, 4H, *J* = 6.74), 1.90 (quint, 4H, *J* = 6.58). Expected mass [H^+^, m/2]: 317.08, observed mass: 317.08 (m/2).

#### 4-azidobutanoic acid (N_3_-COOH)

N_3_-COOH was synthesized based on published procedures and used with minor modifications^43^. Ethyl-4-bromobutryate (36.6 mL, 254 mmol) and sodium azide (25 g, 380 mmol) were dissolved in DMSO (375 mL) and reacted at 55 °C overnight. The reaction was cooled to room temperature and diluted with water (250 mL) and extracted with diethyl ether (3 × 100 mL). The organic layer was washed with brine (250 mL) and dried over Na_2_SO_4_, and concentrated by rotary evaporator to give the intermediate ethyl-4-azidobutanoate as a yellow oil. Yield: 32.5 g, 80%.

Ethyl-4-azidobutanoate (32.5 g, 0.205 mmol) was dissolved in 150 mL of methanol. 1 M sodium hydroxide (aqueous, 250 mL) was added and the mixture was stirred at room temperature for 3 h. Methanol (~ 75 mL) was partially removed by rotary evaporation and concentrated hydrochloric acid (HCl) was added until the pH reached 1. The product was extracted into diethyl ether (3  × 250 mL), dried over Na_2_SO_4_, and the ether was removed to give 4-azidobutanoic acid as a yellow oil. Yield: 27 g, 84%. ^1^H NMR (CDCl_3_, 300 MHz) 6.29 (br s, 1H), 3.51 (q, 2H, *J* = 7.04), 3.37 (t, 2H, *J* = 6.72), 2.45 (quint, 2H, *J* = 7.23), 1.21 (t, 2H, *J* = 7.03).

#### 4-(4-(1-((4-azidobutanoyl)oxy)ethyl)-2-methoxy-5-nitrophenoxy)butanoic acid (N_3_-oNB-COOH)

N_3_-COOH (27 g, 209 mmol) and N,N´-Dicyclohexylcarbodiimide (DCC, 13.8 g, 67 mmol) were mixed under nitrogen in a flame-dried flask. Dry dichloromethane (DCM, 170 mL) was added and the reaction stirred at room temperature for 1 h. Solid urea byproduct was filtered over glass frit and the reaction mixture concentrated by rotary evaporation (filtration step repeated if more byproduct precipitate was observed).

HO-*o*NB-COOH (4-(4-(1-hydroxyethyl)-2-methoxy-5-nitrophenoxy)butanoic acid, 4.2 g, 14 mmol) and 4-dimethylaminopyridine (DMAP, 86 mg, 0.7 mmol) were mixed with the crude anhydride and dissolved in minimal dichloromethane (DCM, 100 mL). Pyridine (1.13 mL, 14 mmol) was added and reaction was stirred under nitrogen overnight until color turned dark brown. Reaction was washed with saturated sodium bicarbonate (NaHCO_3_, 250 mL) and 1 M aqueous HCl and concentrated by rotary evaporation. Reaction was dissolved in 1:1 water:acetone (500 mL) and stirred overnight at room temperature. SAFETY HAZARD: washes with 1 M HCl will generate CO_2_ gas; vent appropriately.

Acetone was removed by rotary evaporation and product extracted into dichloromethane (3 × 250 mL). The organic layer was washed with 1 M HCl, dried over sodium sulfate and concentrated. Product was purified by flash silica column chromatography, 20–40% ethyl acetate in hexanes with 1% acetic acid, product eluted as trailing yellow band giving N_3_-*o*NB-COOH as a yellow oil. Yield: 4.3 g, 71%. ^1^H NMR (CDCl_3_, 300 MHz) δ 7.57 (s, 1H), 7.10 (s, 1H), 6.21 (q, 1H, *J* = 6.44), 4.08 (t, 2H, *J* = 6.47), 3.96 (s, 3H), 3.32 (t, 2H, *J* = 6.84), 2.43 (t, 2H, *J* = 6.21), 2.38 (t, 2H, *J* = 6.34), 1.96 (quint, 2H, *J* = 6.91), 1.76 (t, 2H, *J* = 6.98), 1.58 (d, 3H, *J* = 6.54).

#### 1-(4-(4-((2-(2-(2-azidoethoxy)ethoxy)ethyl)amino)-4-oxobutoxy)-5-methoxy-2-nitrophenyl)ethyl 4-azidobutanoate (oNB)

N_3_-*o*NB-COOH (2.1 g, 5 mmol), 1-[Bis(dimethylamino)methylene]−1H-1,2,3-triazolo[4,5-b]pyridinium 3-oxid hexafluorophosphate (HATU, 950 mg, 2.5 mmol, Sigma Aldrich), and diisopropylamine (DIEA, 3.56 mL, 20 mmol, Chem-Impex International) were dissolved in DMF and stirred for 20 min to prereact. N_3_-TEG-NH_2_ (741 µL, 5.1 mmol, Lumiprobe) was added and the reaction stirred at room temperature for 90 min. The reaction mixture was diluted with ethyl acetate (300 mL), washed with water (3 × 100 mL), dried over magnesium sulfate, and concentrated by rotary evaporation. Product (denoted *o*NB) was purified by silica flash chromatography, 2:1 ethyl acetate:hexanes; collected the last yellow band. Yield: 849 mg, 30%. Overall yield: 18% ^1^H NMR: (CDCl_3_, 300 MHz) δ 7.55 (s, 1H), 7.00 (s, 1H), 6.45 (q, 1H, *J* = 6.35), 6.27 (brd t, 1H), 4.07 (t, 2H, *J* = 5.43), 3.95 (s, 3H), 3.66 (t, 2H, *J* = 4.92), 3.62 (s, 4H), 3.54 (t, 2H, *J* = 5.09), 3.44 (p, 2H, *J* = 5.22), 3.37 (t, 2H, *J* = 4.86), 3.31 (t, 2H, *J* = 6.63), 2.47−2.37 (m, 4H), 2.17 (t, 2H, *J* = 6.69), 1.87 (q, 2H, *J* = 9.15), 1.60 (d, 3H, *J* = 6.39). Expected Mass: [+H] 567.25, observed 567.21.

#### PEG-tetraBCN

Following previously published protocol^43^, PEG-tetraamine (M_n_~20 kDa, 4-arm, 1.13 g, 0.0571 mmol, JenKem Technology USA) and BCN-OSu (100 mg, 0.343 mmol, Sigma Aldrich) were added to a flame-dried scintillation vial under nitrogen. Anhydrous DMF (5 mL) and DIEA (159 uL, 4x) was added and the reaction was stirred under nitrogen overnight at room temperature. Water (50 mL) was added to the reaction mixture which was then dialyzed in SpectraPor dialysis tubing (3k MWCO) overnight at 4 °C. The PEG-tetraBCN was recovered as a powder by lyophilization and resuspended at 10 mM in sterile-filtered PBS for gel formation.

### Quantum yield determination

The quantum yield of photocleavage was determined via kinetic analysis of the cleavage reaction as observed by UV-Vis spectrometry^54^. In brief, the absorbance at a given wavelength for each crosslinker (550 nm for Ru(biq)_2_, 445 nm for Ru(bpy)_2_, and 375 for *o*NB) was tracked as each sample was exposed to light. The data were fit to an equation of the form:

1 $$y={A}_{1}{e}^{-x/{\tau }_{1}}+{A}_{2}{e}^{-x/{\tau }_{2}}+{y}_{0}$$

with two time constants τ_1_ and τ_2_ that give rate constants *k*_*1*_ and *k*_*2*_ according to

2 $${k}_{1}=-\frac{1}{{\tau }_{1}},\,{k}_{2}=-\frac{1}{{\tau }_{2}}$$

The quantum yield (Φ) was extracted from the rate constant for each crosslinker according to

3 $$\Phi=\frac{{k}_{1}{[A]}_{i}{V}_{sample}}{\frac{P}{{E}_{ph}{N}_{A}}}$$

Where:

k_1_ is the rate constant for photolysis

[A]_I_ is the initial concentration of the crosslinker

V_sample_ is the total volume of the sample irradiated

P is the power of the incoming light, measured in Watts ( J sec^−^^1^)

E_ph_ is the energy of the photon, given by $\frac{{hc}}{\lambda }$, where h is Plank’s constant, c is the speed of light in a vacuum, and λ is the wavelength of incident light

N_A_ is Avogadro’s number

### Hydrogel formation

Stock solutions of Rubiq and Rubpy crosslinkers were created at 40 mM in PBS and stored at −80 °C until use. Stock solutions of *o*NB were made at 200 mM in DMSO and stored at −80 °C until use. Hydrogels were formed via SPAAC by mixing PEG-tetraBCN (final concentration of 3 mM) with the diazide crosslinkers (final concentration of 6 mM) with PBS as additional buffer. For hydrogels used in cell culture, an azide-modified RGD peptide (N_3_-GRGDSG-NH_2_) was included (final concentration of 1 mM) and cell media was used in place of PBS.

### Rheometric characterization of hydrogel viscoelasticity

Hydrogels were formed on the rheometer (20 µL, working distance = 0.3 mm) and gelation was observed in a timesweep experiment (angular frequency = 6.28 rad s^−^^1^). Following complete hydrogel formation as indicated by storage modulus plateau, gels were swelled in 0.1 mM NaN_3_ to cap any remaining BCN groups prior to photodegradation. Hydrogels were exposed to light via a quartz bottom plate coupled to a fiber optic light guide from a multiwavelength LED light source.

### Ex vivo degradation through pork belly tissue

Hydrogels were placed in between layers of pork belly purchased from a local butcher (B & E Meats and Seafood, Seattle, WA). For the duration of the experiment, the pork belly and light source was kept in a dark room and covered with moist paper towels to prevent drying. A multiwavelength LED light source was placed directly on the skin and tuned to the maximum power for each wavelength: 617 nm: 350 mW cm^−2^, 530 nm: 314 mW cm^−^^2^, 405 nm: 662 mW cm^−^^2^.

### Mammalian cell culture

All cells were grown to 90% confluence at 37 °C and 5% CO_2_ before use for experiments. 10T1/2 fibroblast-like cells isolated from mouse embryo cells were gifted by Dr. Jennifer Davis (University of Washington). Mouse 10T1/2 fibroblasts were cultured in DMEM with 100 mM penicillin and streptomycin (p/s) and 10% fetal bovine serum (FBS). Fluorescent and non-fluorescent hS5 human stromal cells (from a White, 30-year-old, male) were gifted by Dr. Beverly Torok-Starb (Fred Hutch) (originally purchased from ATCC). hS5s were cultured in RPMI media with 100 mM p/s and 10% FBS. Human bone-marrow derived mesenchymal stem cells (hMSCs) were purchased from Sigma Aldrich. hMSCs were cultured in DMEM (100 mM p/s, 10% FBS) to confluency following thawing and then in MesenPRO medium (Gibco) following the first passage.

### Cell viability following exposure to Rubiq and Rubpy crosslinkers

hS5 and 10T1/2 cells were plated at 1500 cells per well in a white-walled 96-well plate. 24 h after plating, cells were exposed to Rubiq, Rubpy, or Rubiq/Rubpy prereacted with monofunctionalized methoxy-PEG-BCN (M_n_~10 kDa) at a final concentration of 1 mM in the appropriate growth media for 1 h. The media was then replaced with fresh media supplemented with substrate and nLuc enzyme for RealTime Glo assay (Promega). Luminescence was observed over the course of 72 h by plate reader, with an increase in luminescence suggesting an increase in cell proliferation.

### Cell encapsulation in PEG hydrogels

Trypsinized cells (i.e., 10T1/2s, hS5s, hMSCs) were each encapsulated in SPAAC hydrogels at 5 × 10^6^ cells mL^−^^1^ via direct mixing with hydrogel precursors. Hydrogels (10 µL) were formed and incubated at 37 °C and 5% CO_2_ for 30 min before being submerged in the appropriate media (see Mammalian Cell Culture section).

### Encapsulated cell viability

Encapsulated cell viability was assayed by live/dead staining with calcein green and ethidium homodimer (EtHD), respectively. Hydrogels were washed in PBS and incubated in live/dead staining solution (4 μM EtHD, 2 μM calcein) for 1 h prior to confocal imaging. Live/dead cell count was quantified from max intensity projections spanning 40 µm × 50 µm × 50 μm of hydrogel using CellProfiler and normalized to cell viability in TEG-crosslinked hydrogels.

### Released cell viability

Viability of released cells were assessed via flow cytometry. Cell-laden hydrogels were degraded using the appropriate wavelength of light (10 mW cm^−2^) into 1 mL sterile PBS. Cells were collected by pelleting and resuspended in calcein/EtHD solution for 15 min before storing on ice and FACS counting.

### EdU assay for cell proliferation

Cell-laden hydrogels were cast in glass-bottom 35 mm petri dishes. 24 h after casting, hydrogels were degraded with the appropriate wavelength of light (10 mW cm^−^^2^ for all wavelengths) into a minimum of PBS (200–300 μL). Cells were allowed to attach to the surface for 2 h before the appropriate growth media supplemented with 10 μM EdU was added. After 72 h cells were fixed, permeabilized, and new DNA with incorporated EdU was tagged with AF-568-azide (Lumiprobe) via copper-catalyzed azide-alkyne cycloaddition (ClickIT EdU assay, Invitrogen). Nuclei were stained with Hoescht and fluorescently imaged. CellProfiler was used to count the total number of nuclei and number of EdU^+^ nuclei.

### Caspase 3 assay for apoptosis

hS5 and 10T1/2 cells were plated at 1500 cells per well in a white-walled 96-well plate. 24 h after plating, cells were exposed to Rubiq, Rubpy, or Rubiq/Rubpy prereacted with mono-PEG-BCN at a final concentration of 1 mM in the appropriate growth media for 3 h. 24 h after exposure, caspase 3 levels were determined via the ApoTox Glo assay, where an increase in luminescence indicates increased levels of caspase 3 and the presence of apoptotic cells (Promega).

### Photorelease of human stromal cells from gels for flow cytometry

hS5 stromal cells stably expressing BFP, GFP, and mCherry were encapsulated in PEG-tetraBCN hydrogels crosslinked with the appropriate photocleavable crosslinker (Rubiq with hS5-mCherry^+^, Rubpy with hS5-GFP^+^, and *o*NB with hS5-BFP^+^). All three hydrogels were cast in the same tissue culture dish, allowed to form for 30 min, then incubated in RPMI media for 24 h.

Hydrogels were transferred to PBS and exposed to 617 nm, 530 nm, or 405 nm light (10 mW cm^−2^, until hydrogel degradation was observed) and the resulting cell suspension was collected and analyzed by flow cytometry. Any hydrogels left intact after irradiation were imaged using confocal fluorescent microscopy.

### Statistics & reproducibility

Unless indicated otherwise, all experiments were performed with *N*  =  3 replicates (3 biological replicates). No statistical method was used to predetermine sample size. No data were excluded from the analyses.

### Reporting summary

Further information on research design is available in the Nature Portfolio Reporting Summary linked to this article.

### Supplementary information

Supporting Information

Reporting Summary

### Source data

Source Data

## Data Availability

All data supporting the findings of this study are available within the article and its supplementary files. Any additional requests for information can be directed to, and will be fulfilled by, the lead contact. Source data are provided with this paper.
