# Supplementary material for: Tricolor visible wavelength-selective photodegradable hydrogel biomaterials
Source: Nat Commun. 2023 Aug 29;14:5250. doi: 10.1038/s41467-023-40805-w (PMC10462736; doi:10.1038/s41467-023-40805-w)
Supplement: Supplementary file 1 — Supporting Information [file 41467_2023_40805_MOESM1_ESM.pdf]

Supplementary Information for

Tricolor Visible Wavelength-selective Photodegradable Hydrogel Biomaterials

Teresa L. Rapp & Cole A. DeForest*

*Corresponding author. Email: profcole@uw.edu

Contents

Materials and instrumentation 4

Supplementary Method 1. Synthetic schemes and methods 5

4-azidobutanoic acid (N_3-COOH) 5

4-(4-(1-((4-azidobutanoyl)oxy)ethyl)-2-methoxy-5-nitrophenoxy)butanoic acid (N_3-*o*NB-OH) 5

1-(4-(4-((2-(2-(2-azidoethoxy)ethoxy)ethyl)amino)-4-oxobutoxy)-5-methoxy-2-nitrophenyl)ethyl 4-azidobutanoate (*o*NB) 6

4-azidobutyronitrile 7

Ru(biquinoline)$_2$Cl$_2$ (Ru(biq)$_2$Cl$_2$) 7

Ru(biq)$_2$(4-azidobutyronitrile)$_2$[Cl$_2$] (Rubiq) 8

Ru(bpy)$_2$(4-azidobutyronitrile)$_2$[Cl$_2$] (Rubpy) 8

Supplementary Figure 1. ^{1}H NMR of Rubiq 10

Supplementary Figure 2. ESI-MS analysis of Rubiq 12

Supplementary Figure 3. ^{1}H NMR analysis of Rubpy 13

Supplementary Figure 4. ESI-MS analysis of Rubpy 14

Supplementary Figure 5. ^{1}H NMR analysis of *o*NB 15

Supplementary Figure 6. ESI-MS of *o*NB 16

Supplementary Figure 7. Jablonski diagram of Ru(II) electronic states 17

Supplementary Figure 8. ^{1}H NMR analysis of Rubiq ligand exchange 18

Supplementary Figure 9. ^{1}H NMR analysis of Rubpy ligand exchange 19

Supplementary Figure 10. ESI-MS analysis of Rubiq photolysis 20

Supplementary Figure 11. ESI-MS analysis of Rubpy ligand exchange 22

Supplementary Figure 12. Singlet oxygen generation during crosslinker photolysis 24

Supplementary Figure 13. Hydrogel stability at 37 °C and room temperature 25

Supplementary Figure 14. Rheological analysis for TEG-doped hydrogel formation 26

Supplementary Figure 15. Viability of hS5s following exposure to Rubiq or Rubpy in vitro 27

Supplementary Figure 16. Viability of 10T1/2 fibroblasts following exposure to Rubiq and Rubpy in vitro 28

Supplementary Figure 17. Caspase 3 detection in hS5 cells following exposure to Rubiq or Rubpy in vitro 29

Supplementary Figure 18. Caspase 3 detection in 10T1/2 cells following exposure to Rubiq or Rubpy in vitro 30

Supplementary Figure 19. Viability and proliferation of hMSCs following hydrogel encapsulation and release 31

Supplementary Figure 20. Viability and proliferation of hS5 cells following hydrogel encapsulation and release 32

Supplementary Figure 21. Gating strategy for measuring live/dead cell populations via flow cytometry. 33
Supplementary Figure 22. Gating strategy for cell population quantification via flow cytometry 34
Supplementary references 35

Materials and instrumentation

Chemical reagents and solvents were purchased from either Sigma-Aldrich or Fisher Scientific and used as received. Synthetic chemical reactions were performed in red light and stirred with a Teflon-coated magnetic stir bar unless otherwise noted. Solvents were removed in vacuo with a Büchi Rotovapor R-3 equipped with a V-700 vacuum pump and V-855 vacuum controller and a Welch 1400 DuoSeal Belt-Drive high vacuum pump. ^1H nuclear magnetic resonance (NMR) data was collected at 298 K on Bruker instruments and chemical shifts are reported relative to tetramethylsilane (TMS, $\delta = 0$). Lyophilization was performed on a LABCONCO FreeZone 2.5 Plus freeze-dryer equipped with a LABCONCO rotary vane 117 vacuum pump. High-resolution mass spectrometry (HRMS) was performed on a Thermo Linear Trap Quadrupole Orbitrap Xcalibur 2.0 DS.

Rheological measurements were performed on an Anton Paar MCR301 equipped with a quartz bottom plate for light exposure and PP08 measuring plate. Confocal microscopy was performed on a Leica SP8X. Mammalian cell culture was performed in a NuAire LabGard ES NU-437 Class II Type A2 Biosafety Cabinet. Cells were maintained in a Sanyo inCu saFe® MCO-17AC incubator at 37 °C and 5% CO2. Flow cytometry was performed at the University of Washington Pathology Flow Cytometry Core Facility on a BD Biosciences LSR II Flow Cytometer.

Photolysis was performed with a Mightext BioLED light source (BLS series) equipped with a fiber optic bundle light guide (non-collimated) for illumination of samples/cell culture. Light power was determined using an OmniCure R2000 radiometer.

Supplementary Method 1. Synthetic schemes and methods

4-azidobutanoic acid (N_3-COOH)

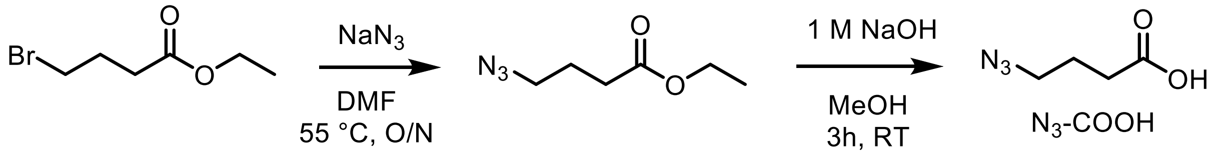

N_3-COOH was synthesized based on published procedures and used with minor modifications[1]. Ethyl-4-bromobutryate (36.6 mL, 254 mmol) and sodium azide (25 g, 380 mmol) were dissolved in DMSO (375 mL) and reacted at 55 °C overnight. The reaction was cooled to room temperature and diluted with water (250 mL) and extracted with diethyl ether (3x100 mL). The organic layer was washed with brine (250 mL) and dried over Na_2SO_4, and concentrated by rotary evaporator to give the intermediate ethyl-4-azidobutanoate as a yellow oil. Yield: 32.5 g, 80%.

Ethyl-4-azidobutanoate (32.5 g, 0.205 mmol) was dissolved in 150 mL of methanol. 1M sodium hydroxide (aqueous, 250 mL) was added and the mixture was stirred at room temperature for 3 hours. Methanol (~75 mL) was partially removed by rotary evaporation and concentrated hydrochloric acid (HCl) was added until the pH reached 1. The product was extracted into diethyl ether (3 x 250 mL), dried over Na_2SO_4, and the ether was removed to give 4-azidobutanoic acid as a yellow oil. Yield: 27 g, 84%. ^{1}H NMR ($CDCl_3$, 300 MHz) 6.29 (br s, 1H), 3.51 (q, 2H, *J*=7.04), 3.37 (t, 2H, *J*=6.72), 2.45 (quint, 2H, *J*=7.23), 1.21 (t, 2H, *J*=7.03)

4-(4-(1-((4-azidobutanoyl)oxy)ethyl)-2-methoxy-5-nitrophenoxy)butanoic acid (N_3-*o*NB-OH)

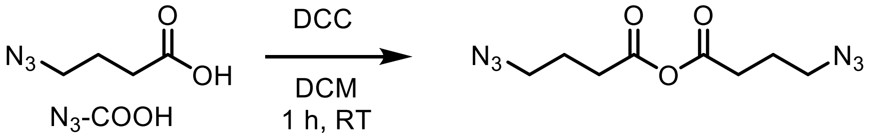

N_3-COOH (27 g, 209 mmol) and N,N′-Dicyclohexylcarbodiimide (DCC, 13.8 g, 67 mmol) were mixed under nitrogen in a flame-dried flask. Dry dichloromethane (DCM, 170 mL) was added and the reaction stirred at room temperature for 1 hour. Solid urea byproduct was filtered over glass frit and the reaction mixture concentrated by rotary evaporation (filtration step repeated if more byproduct precipitate was observed).

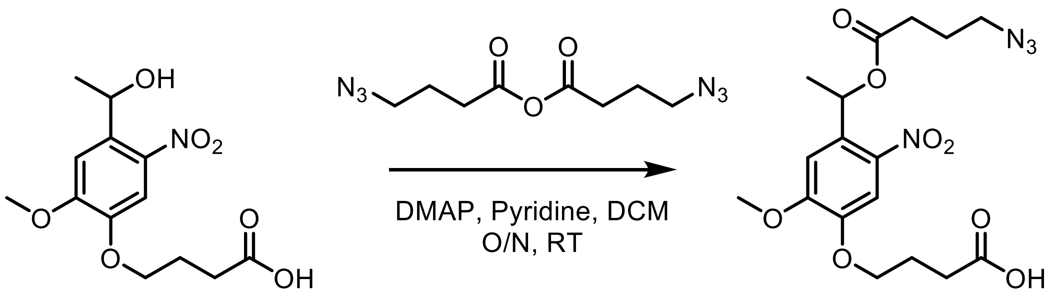

HO-oNB-COOH N_3-oNB-COOH

HO-*o*NB-COOH (4-(4-(1-hydroxyethyl)-2-methoxy-5-nitrophenoxy)butanoic acid, 4.2 g, 14 mmol) and 4-dimethylaminopyridine (DMAP, 86 mg, 0.7 mmol) were mixed with the crude anhydride and dissolved in minimal dichloromethane (DCM, 100 mL). Pyridine (1.13 mL, 14 mmol) was added and reaction was stirred under nitrogen overnight until color turned dark brown. Reaction was washed with saturated sodium bicarbonate ($NaHCO_3$, 250 mL) and 1M aqueous HCl and concentrated by rotary evaporation. Reaction was dissolved in 1:1 water:acetone (500 mL) and stirred overnight at room temperature.

Acetone was removed by rotary evaporation and product extracted into dichloromethane (3 x 250 mL). The organic layer was washed with 1M HCl, dried over sodium sulfate and concentrated. Product was purified by flash silica column chromatography, 20-40% ethyl acetate in hexanes with 1% acetic acid, product eluted as trailing yellow band giving N_3-*o*NB-COOH as a yellow oil. Yield: 4.3 g, 71%. ^{1}H NMR ($CDCl_3$, 300 MHz) δ 7.57 (s, 1H), 7.10 (s, 1H), 6.21 (q, 1H, *J*=6.44), 4.08 (t, 2H, *J*=6.47), 3.96 (s, 3H), 3.32 (t, 2H, *J*=6.84), 2.43 (t, 2H, *J*=6.21), 2.38 (t, 2H, *J*=6.34), 1.96 (quint, 2H, *J*=6.91), 1.76 (t, 2H, *J*=6.98), 1.58 (d, 3H, *J*=6.54).

1-(4-(4-((2-(2-(2-azidoethoxy)ethoxy)ethyl)amino)-4-oxobutoxy)-5-methoxy-2-nitrophenyl)ethyl 4-azidobutanoate (*o*NB)

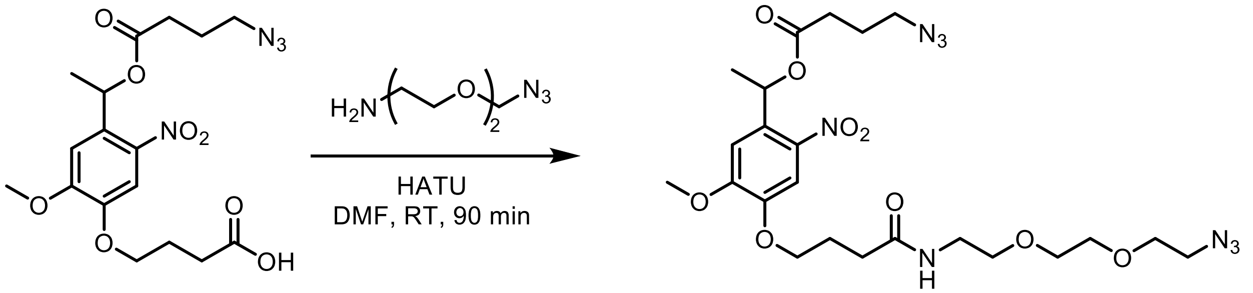

N_3-oNB-COOH

N_3-*o*NB-COOH (2.1 g, 5 mmol), 1-[Bis(dimethylamino)methylene]-1H-1,2,3-triazolo[4,5-b]pyridinium 3-oxid hexafluorophosphate (HATU, 950 mg, 2.5 mmol), and diisopropylamine (DIEA, 3.56 mL, 20 mmol) were dissolved in DMF and stirred for 20 min to prereact. N_3-TEG-NH_2 (741 µL, 5.1 mmol) was added and the reaction stirred at room temperature for 90 min. The reaction mixture was diluted with ethyl acetate (300 mL), washed with water (3 x 100 mL), dried over magnesium sulfate, and concentrated by rotary evaporation. Product (denoted ***o*NB**) was purified by silica flash chromatography, 2:1 ethyl acetate:hexanes; collected the last yellow band. Yield: 849 mg, 30%. Overall yield: 18% ^{1}H NMR: ($CDCl_3$, 300 MHz) δ 7.55 (s, 1H), 7.00 (s, 1H), 6.45 (q, 1H, *J*=6.35), 6.27 (brd t, 1H), 4.07 (t, 2H, *J*=5.43), 3.95 (s, 3H), 3.66 (t, 2H, *J*=4.92), 3.62 (s, 4H), 3.54 (t, 2H, *J*=5.09), 3.44 (p, 2H, *J*=5.22), 3.37 (t, 2H, *J*=4.86), 3.31 (t, 2H,

J=6.63), 2.47-2.37 (m, 4H), 2.17 (t, 2H, J=6.69), 1.87 (q, 2H, J=9.15), 1.60 (d, 3H, J=6.39). Expected Mass: [+H] 567.25, observed 567.21.

4-azidobutyronitrile

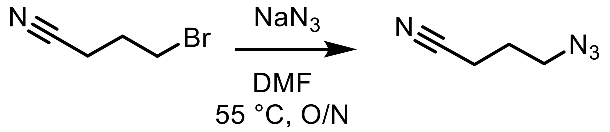

4-bromobutyronitrile (1 mL, 10 mmol) and sodium azide (1.3 g, 20 mmol) were dissolved in dimethylsulfoxide (DMSO, 15 mL) and stirred overnight at 55 °C. The reaction was cooled to room temperature, diluted with water to 50 mL, extracted with diethyl ether (3x 50 mL), dried over Na_2SO_4, and the ether was removed under vacuum to give the product as a light-yellow oil. SAFTEY HAZARD: 4-azidobutyronitrile is isolated with DMSO in the final form. It should never be fully dried, as it is an explosive small molecule azide when concentrated. Yield: 1.017 g, 92%. ^{1}H NMR: ($CDCl_3$, 300 MHz) δ 3.50 (t, 2H, J=6.34), 2.48 (t, 2H, J=7.05), 1.92 (quint, 2H, J=12.96).

Ru(biquinoline)$_2$Cl$_2$ (Ru(biq)$_2$Cl$_2$)

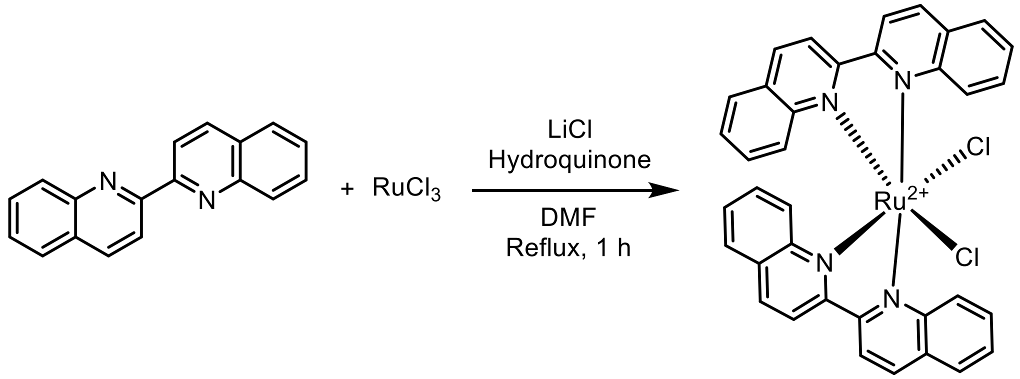

$RuCl_3$ (anhydrous, 99.96% trace metal basis, 400 mg, 1.93 mmol), hydroquinone (444 mg, 4 mmol) and LiCl (480 mg, 11.3 mmol) were suspended in dimethylformamide (DMF, 10 mL) and bubbled with N_2 for 15 min. 2,2'-biquinoline (1 g, 4 mmol) was added, and reaction was heated to 130 °C for 1 h until color appeared forest green, then cooled to room temperature. Reaction was added dropwise to 800 mL deionized water and filtered to collect a dark green-black solid. Solid was dissolved in dichloromethane (200 mL), solvent was reduced to 100 mL under vacuum, then added to diethyl ether (400 mL) and filtered to collect Ru(biq)$_2$Cl$_2$ as a dark green solid. Product was used without further purification. Yield: 805 mg, 61%.

Ru(biq)$_2$(4-azidobutyronitrile)$_2$[Cl$_2$] (Rubiq)

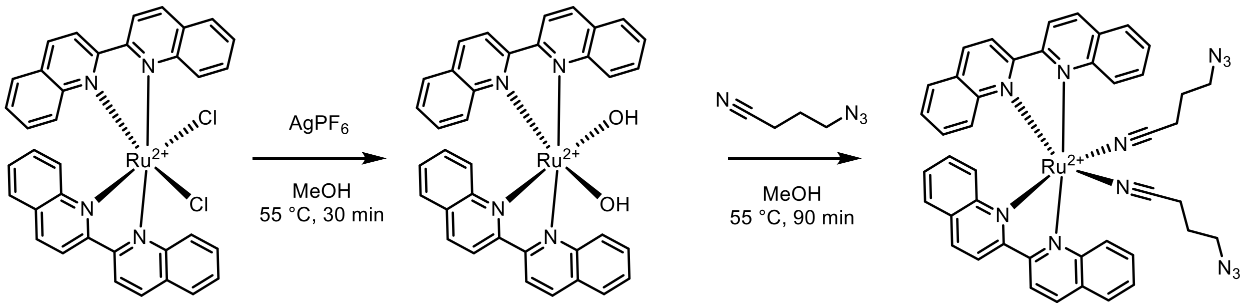

$Ru(biq)_2Cl_2$ (250 mg, 0.365 mmol) and silver hexafluorophosphate (275 mg, 1.08 mmol) were dissolved in methanol (40 mL, stored over molecular sieves) and stirred at 55 °C in the dark until reaction turned blue and white silver chloride powder was observed (15 min). 4-azidobutyronitrile (100 µL, 0.945 mmol) was added, and reaction stirred at 55 °C for 1 hour until color turned pink. Reaction was cooled to room temperature, filtered to remove silver chloride, and methanol was removed by rotary evaporation. $Ru(biq)_2$(4-azidobutyronitrile)$_2$ was purified by silica flash chromatography with 1:4 acetonitrile:dichloromethane as the eluent.

$Ru(biq)_2$(4-azidobutyronitrile)$_2[PF_6]_2$ was converted to the chloride salt by anion-exchange resin. Rubiq$[PF_6]_2$ was passed over Amberlite IRA-410 (Cl Form) resin using methanol as the eluent, giving $Ru(biq)_2$(4-azidobutyronitrile)$_2[Cl]_2$ as the water-soluble form (denoted **Rubiq**). Yield: 159 mg, 48%. Overall yield: 27% ^1H NMR: (CD_3CN, 300 MHz) δ 9.14 (broad s, 2H), 8.68 (d, 2H, *J*=8.69), 8.36 (d, 2H, *J*=7.99), 8.31 (d, 2H, *J*=8.09), 8.21 (broad s, 2H), 8.02 (t, 2H, *J*=7.27), 7.91 (d, 2H, *J*=7.84), 7.50 (t, 2H, *J*=7.04), 6.87 (t, 2H, *J*=7.17), 6.75 (d, 2H, *J*=8.4), 3.08 (t, 4H, *J*=6.42), 2.75 (t, 4H, *J*=7.25), 1.65 (quint, 4H, *J*=6.57). Expected mass [H^+, m/2]: 417.11, observed mass: 417.1125 (m/2).

Ru(bpy)$_2$(4-azidobutyronitrile)$_2$[Cl$_2$] (Rubpy)

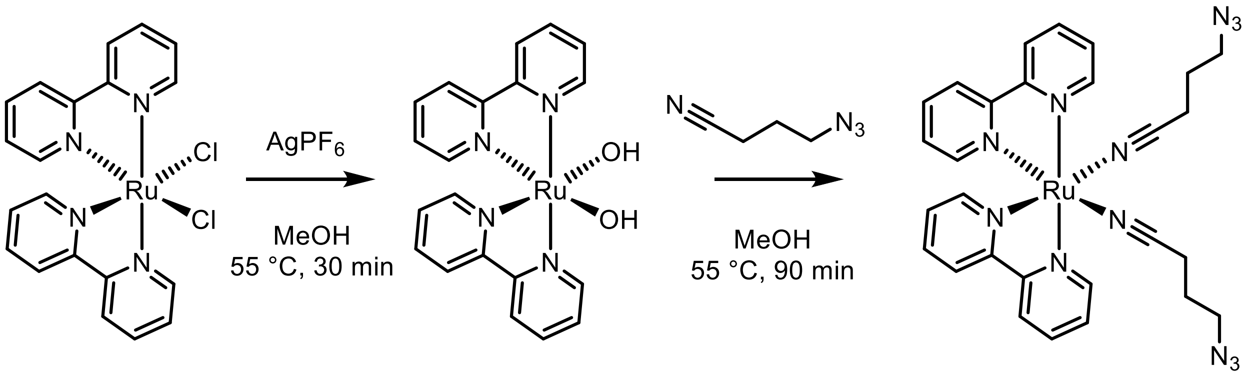

Ru(bipyridine)$_2Cl_2$•$2H_2O$ (200 mg, 0.38 mmol) and silver hexafluorophosphate (212 mg, 0.83 mmol) were dissolved in methanol (stored over molecular sieves, 40 mL) and stirred at 55 °C for 15 minutes. 4-azidobutyronitrile (390 µL, 3.7 mmol) was added and the reaction stirred at 55 °C for 2 hours until color changed from red to orange. Reaction mixture was cooled to room temperature and filtered to remove silver chloride, then concentrated by rotary evaporation. The product (denoted **Rubpy**) was purified by silica flash chromatography with 4:1 dichloromethane:acetonitrile as the eluent, collecting the major yellow band. Yield: 22 mg, 84%. Overall yield: 77% ^1H NMR: (CD_3CN, 300 MHz) δ 9.61 (d, 2H, *J*=4.95), 8.83 (d, 2H,

J=8.07), 8.69 (d, 2H, *J*=8.10), 8.43 (t, 2H, *J*=7.16), 8.12 (t, 2H, *J*=7.16), 8.00 (t, 2H, *J*=6.00), 7.93 (d, 2H, *J*=5.70), 7.46 (t, 2H, *J*=6.00), 3.37 (t, 4H, *J*=6.33), 3.00 (t, 4H, *J*=6.74), 1.90 (quint, 4H, *J*=6.58). Expected mass [H^+, m/2]: 317.08, observed mass: 317.08.

Supplementary Figure 1. ^{1}H NMR of Rubiq

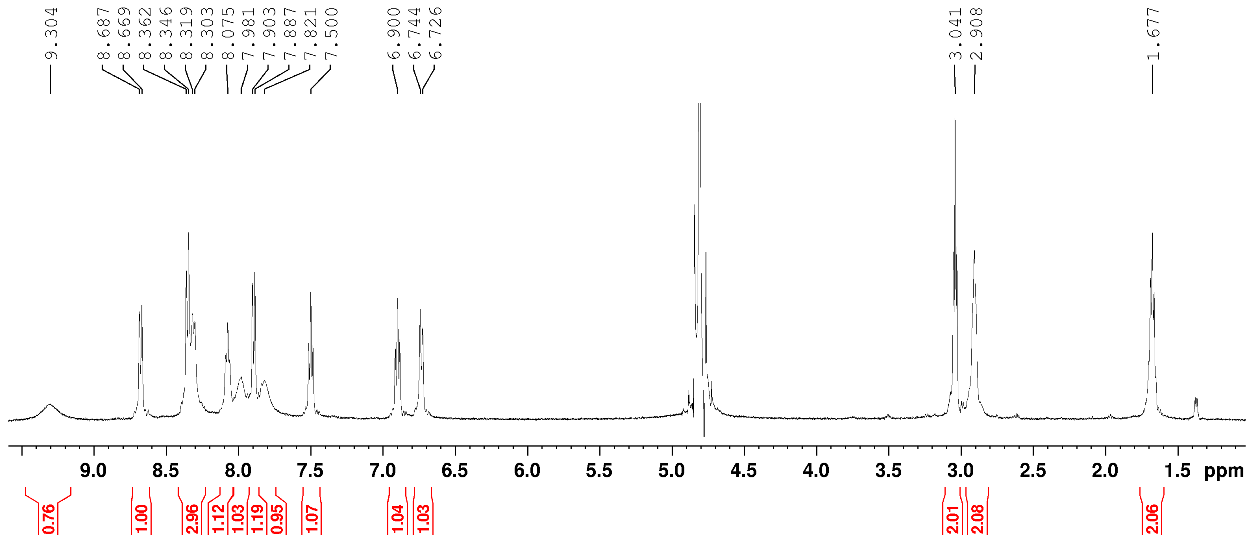

^{1}H NMR in D_2O (500 MHz).

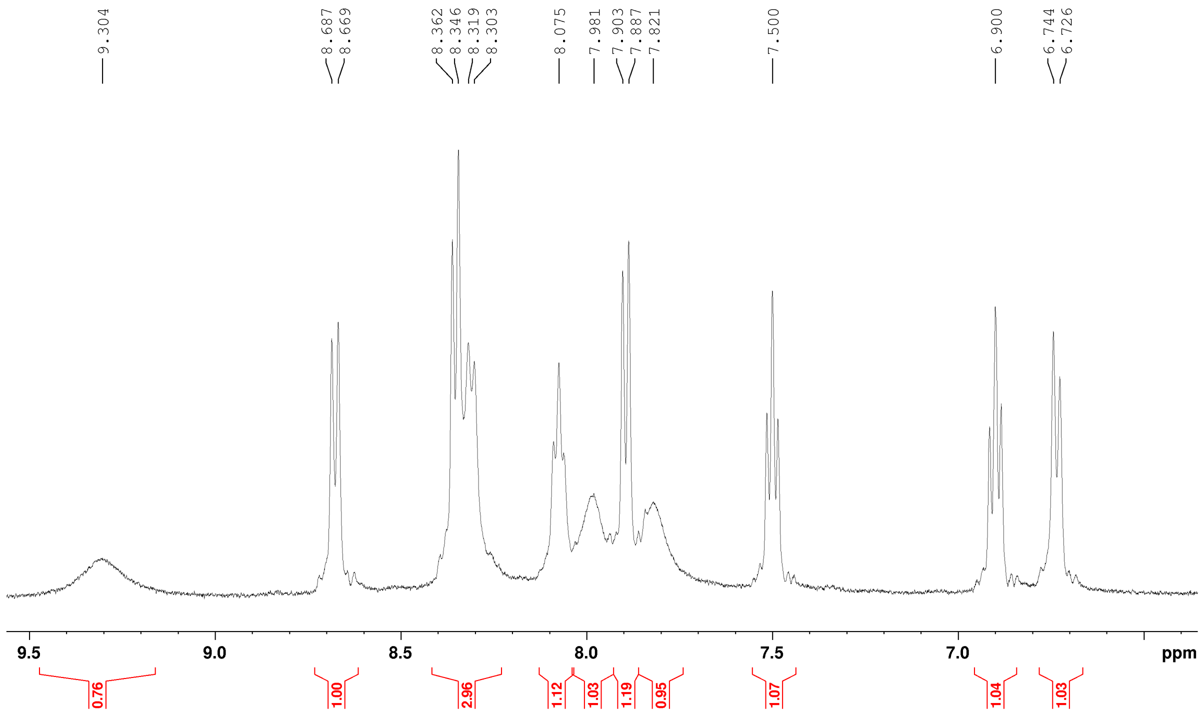

Aromatic region zoom.

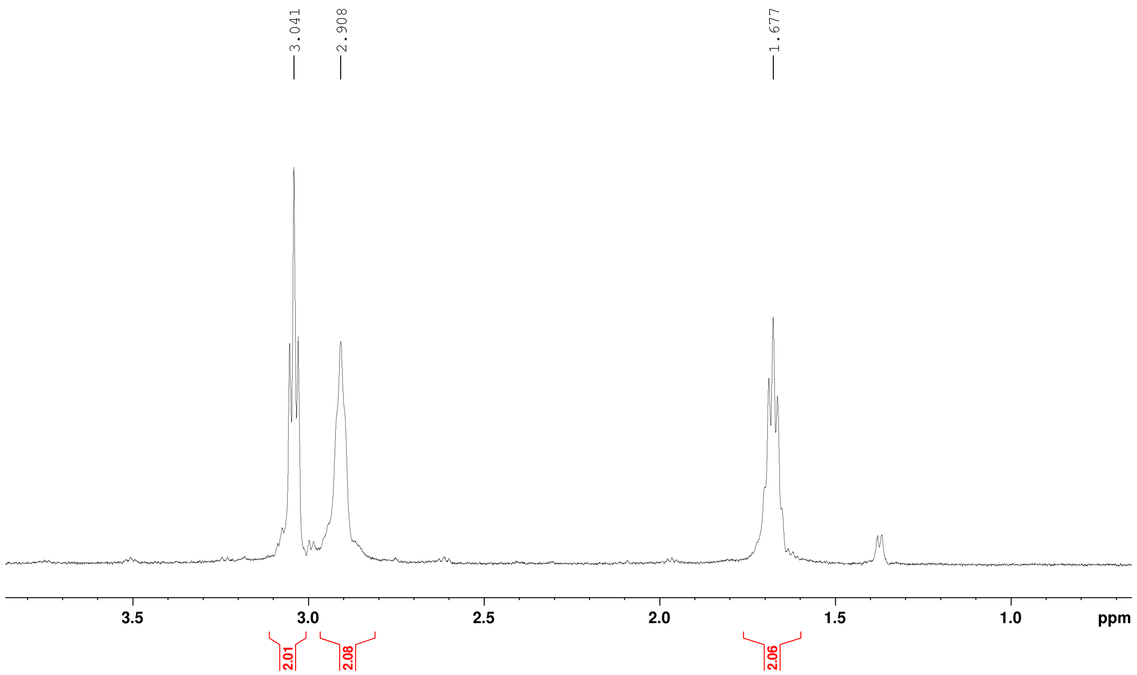

Aliphatic region zoom.

Supplementary Figure 2. ESI-MS analysis of Rubiq

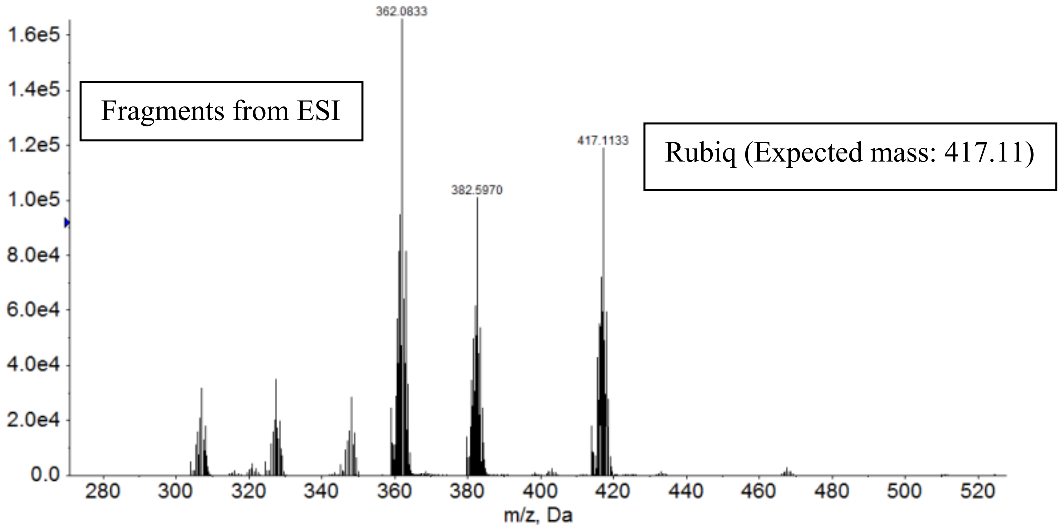

Sample prepared in DI H_2O, direct inject with solvent mixture of 1:1 H_2O:acetonitrile.

Supplementary Figure 3. ^{1}H NMR analysis of Rubpy

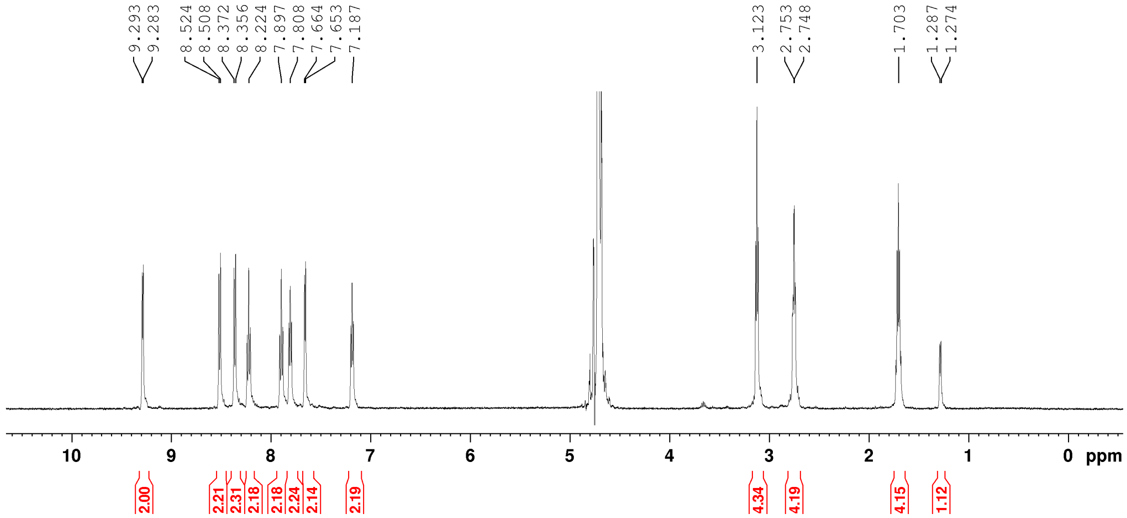

Full ^{1}H NMR in D_2O (500 MHz).

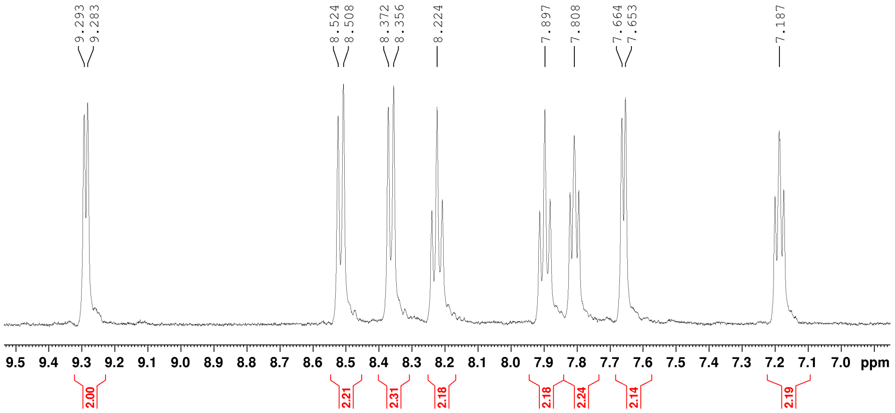

Aromatic region zoom.

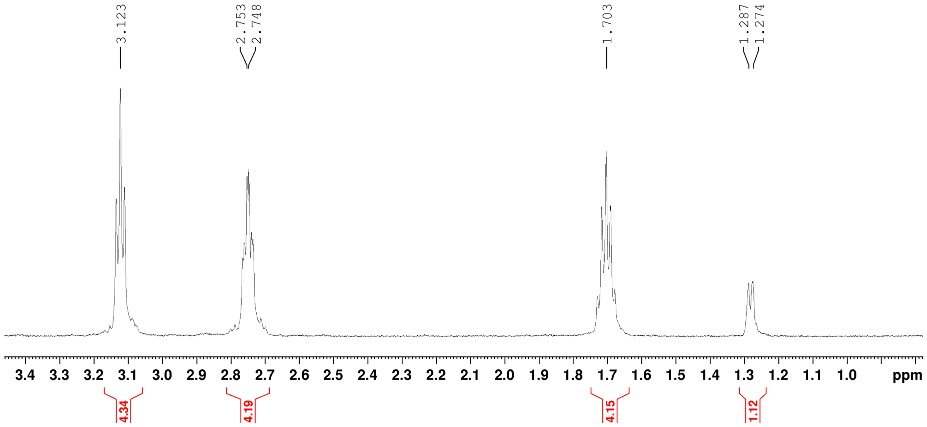

Aliphatic region zoom.

Supplementary Figure 4. ESI-MS analysis of Rubpy

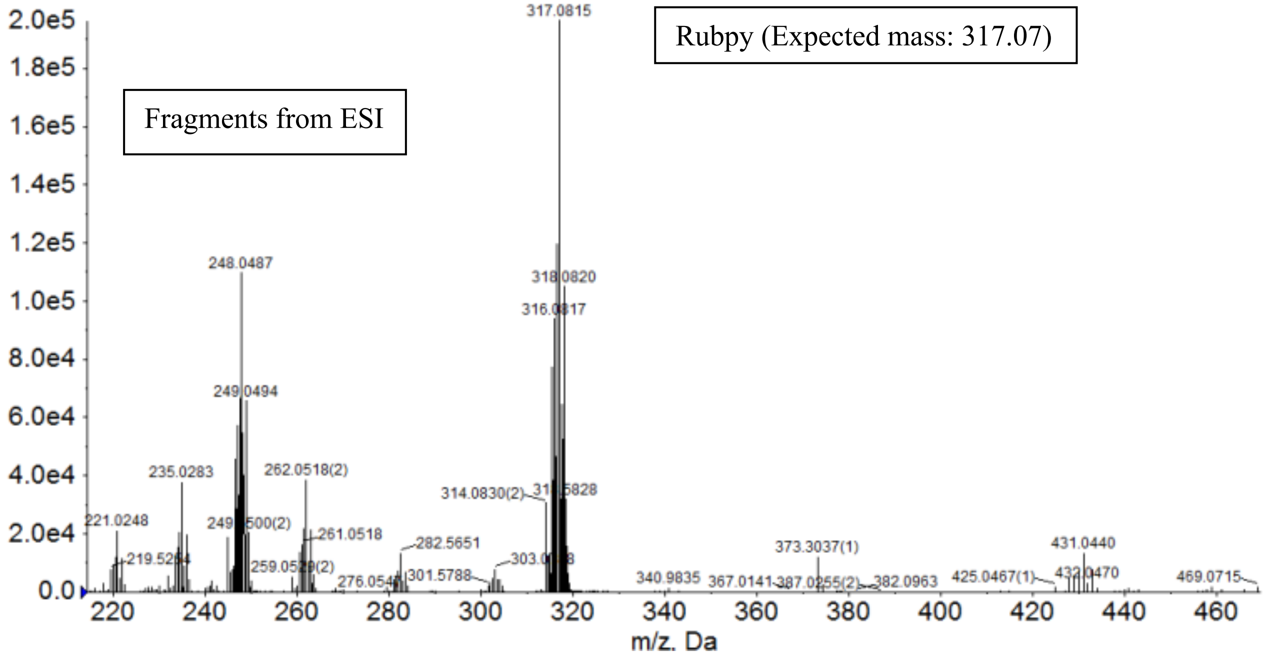

Sample prepared in DI H_2O, direct inject with solvent mixture of 1:1 H_2O:acetonitrile.

Supplementary Figure 5. ^{1}H NMR analysis of *o*NB

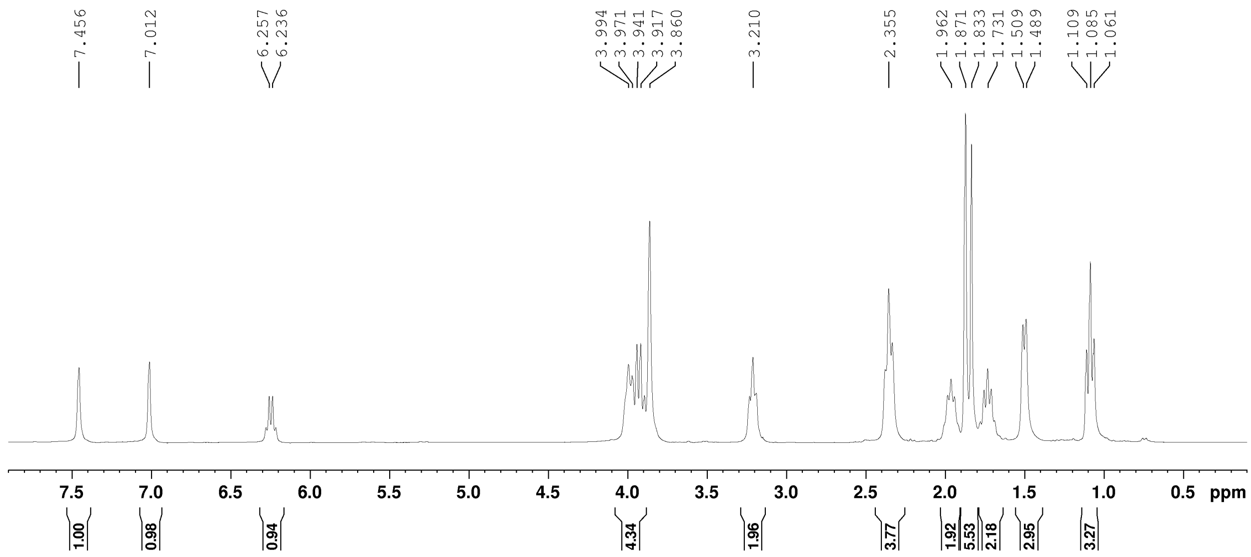

^{1}H NMR (500 MHz) taken in $CDCl_3$.

Supplementary Figure 6. ESI-MS of *o*NB

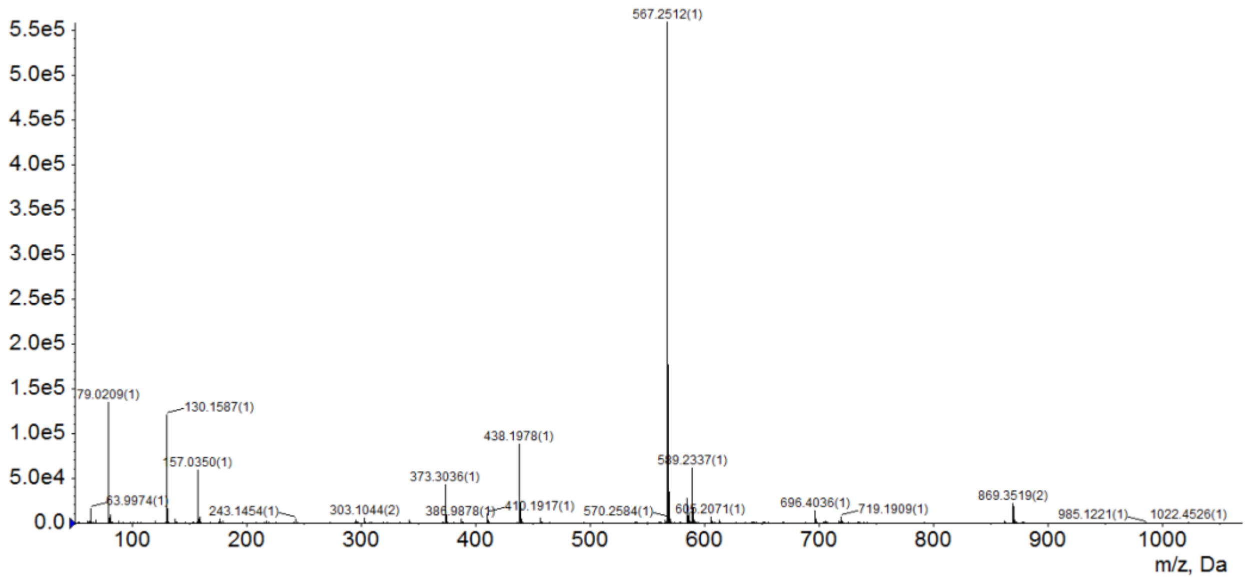

Supplementary Figure 7. Jablonski diagram of Ru(II) electronic states

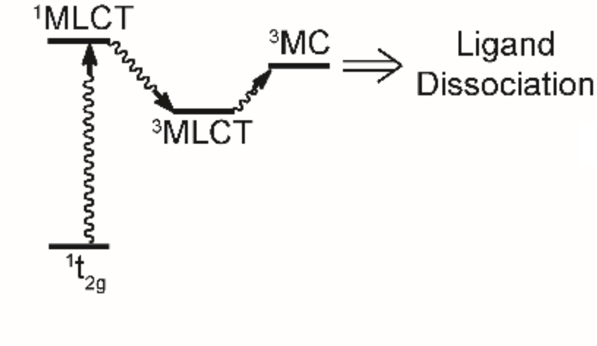

Electronic states responsible for ligand dissociation and exchange with solvent in ruthenium (II) complexes.

Supplementary Figure 8. ^{1}H NMR analysis of Rubiq ligand exchange

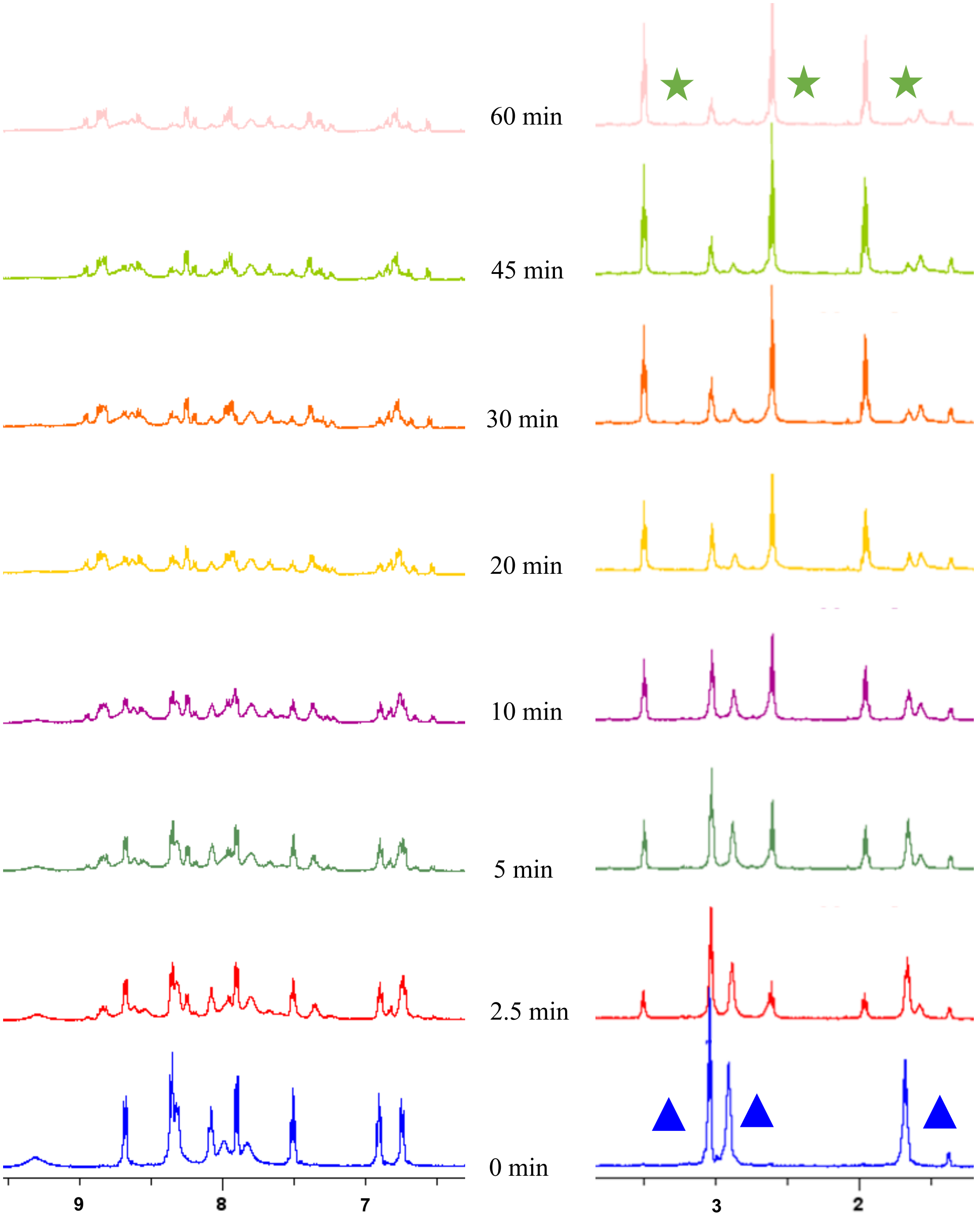

A 5 mM sample of Rubiq was dissolved in D_2O and exposed to 617 nm light (10 mW cm^{-2}) with constant mixing. The sample was probed by ^{1}H NMR at various time points to determine intermediate photolysis products. The complete loss of peaks corresponding to coordinated 4-azidobutyronitrile (blue triangle, 1.68, 2.91, 3.04 ppm) and increase of a new set of peaks (green star, 1.95, 2.60, 3.50 ppm) indicates exchange of both azide-bearing ligands beginning in the first minutes of irradiation. Loss of the broad singlet at 9.30 ppm also suggests gradual loss of one biquinoline ligand after extended irradiation.

Supplementary Figure 9. ^{1}H NMR analysis of Rubpy ligand exchange

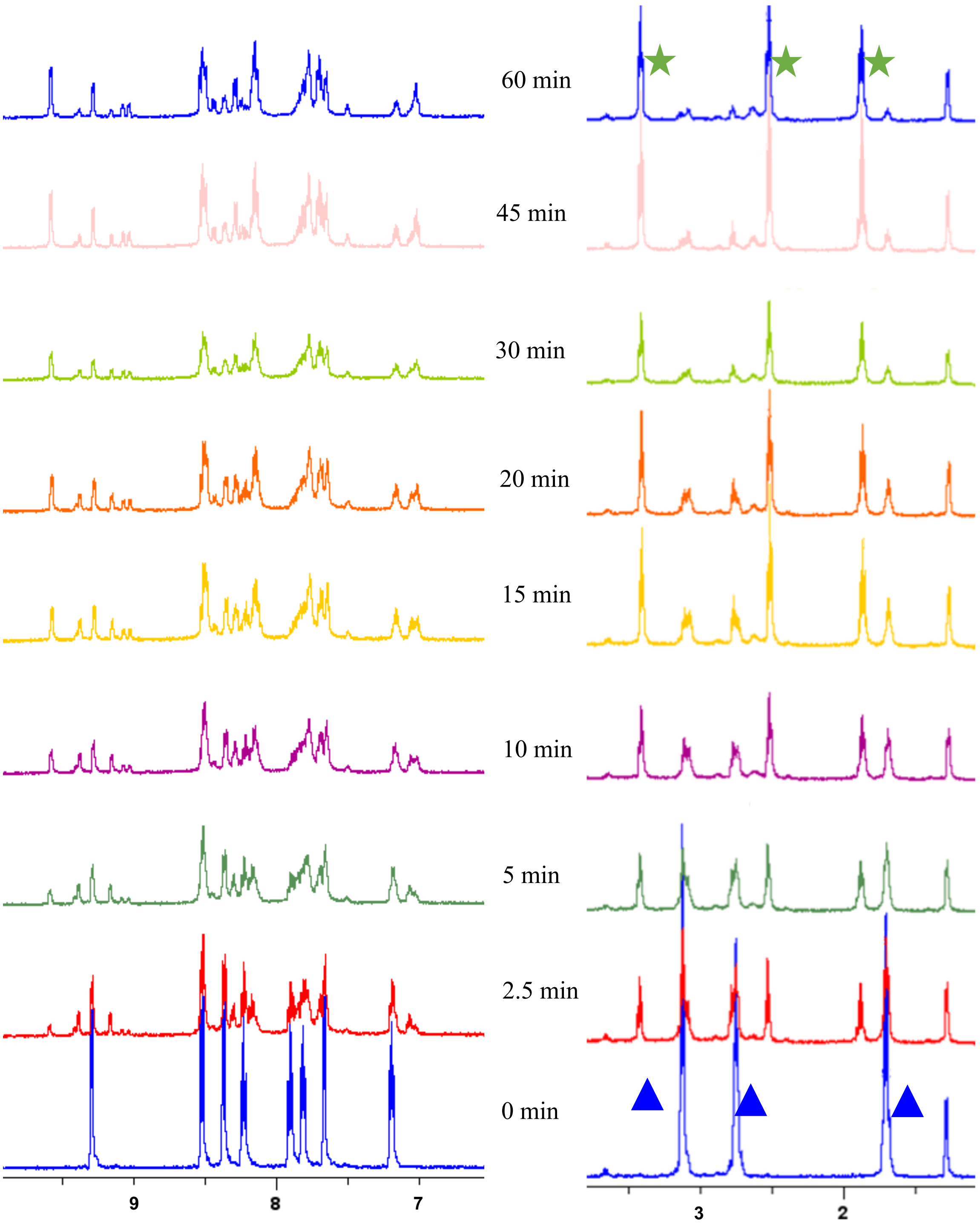

Rubpy (5 mM in D_2O) and exposed to 530 nm light (7.8 mW/cm^2) with constant mixing. The sample was probed by ^{1}H NMR at various time points to determine intermediate photolysis products. The complete loss of peaks corresponding to coordinated 4-azidobutyronitrile (blue triangle, 1.70, 2.75, and 3.13 ppm) and increase of a new set of peaks (green star, 1.87, 2.52, 3.41 ppm) indicates exchange of both azide-bearing ligands beginning in the first minutes of irradiation.

Supplementary Figure 10. ESI-MS analysis of Rubiq photolysis

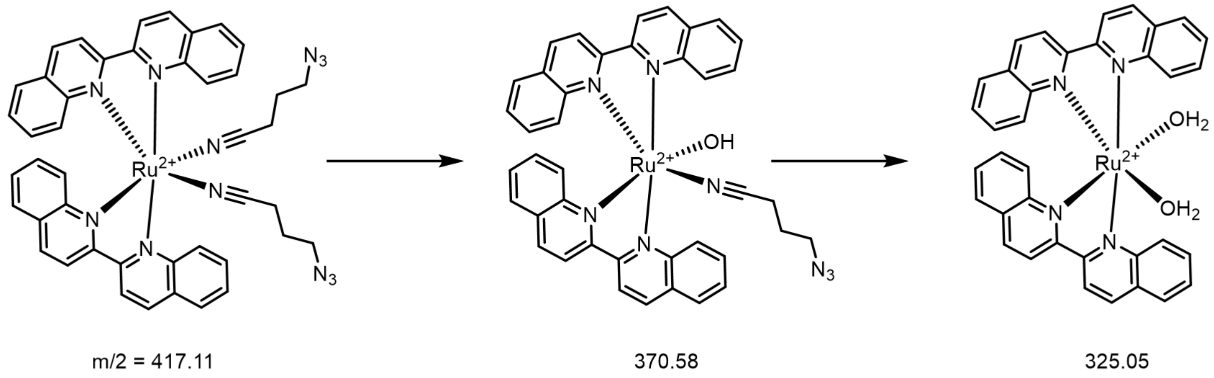

0 min

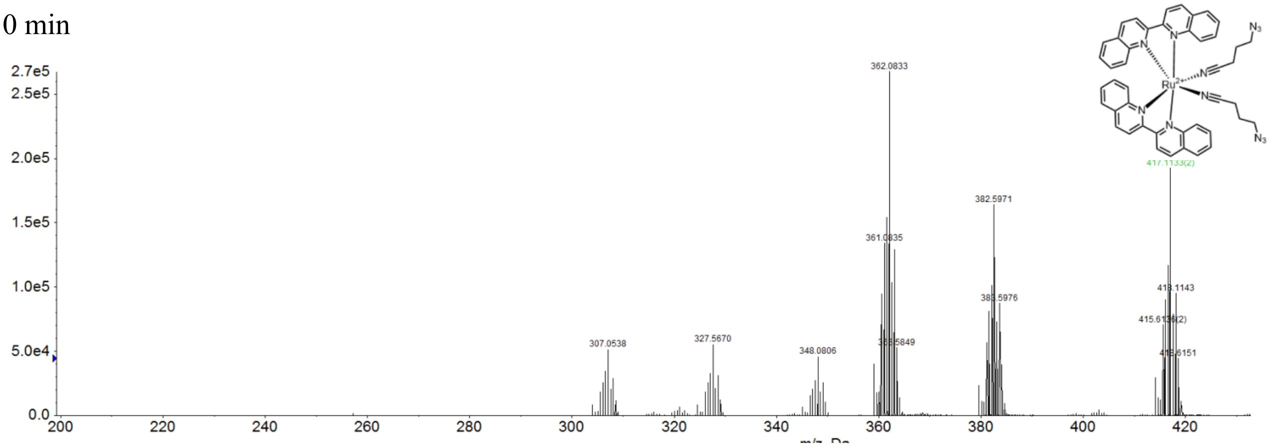

5 min

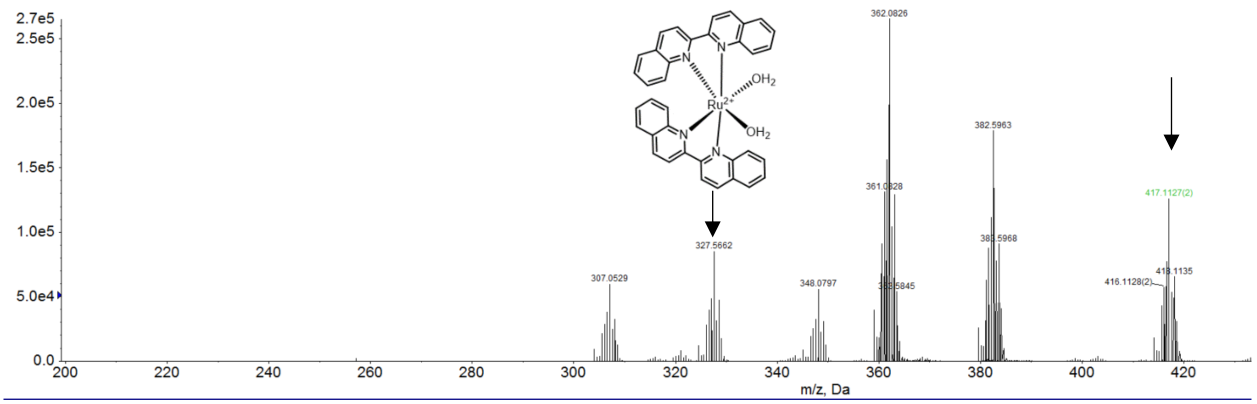

10 min

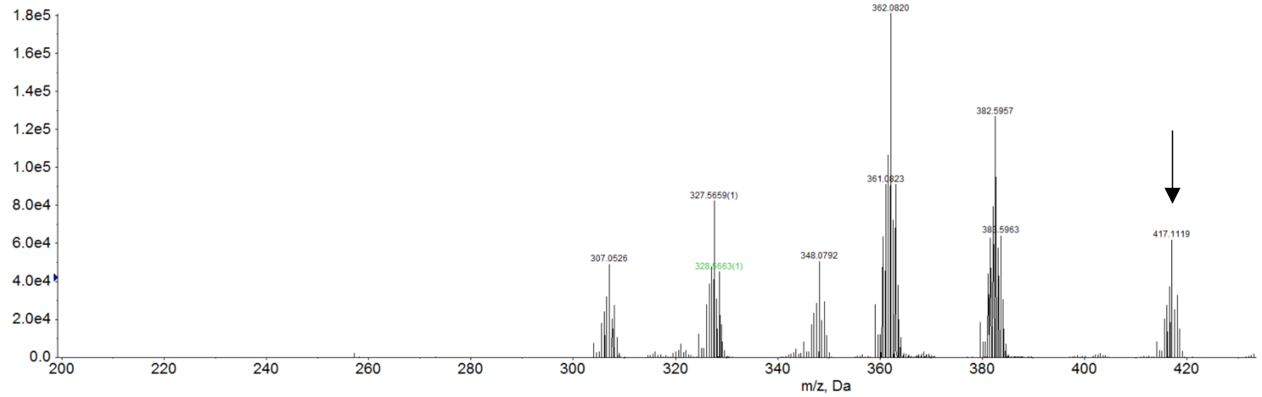

30 min

m/z = 256.10

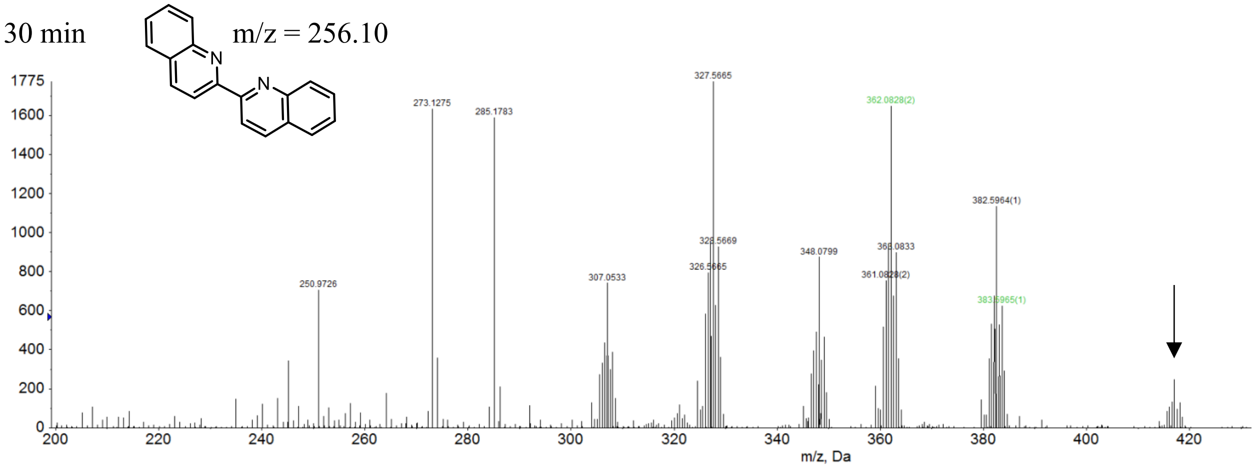

Rubiq (5 mM in H_2O) and exposed to 617 nm light (10 mW/cm^2) with constant mixing. Samples were removed at 5, 10, and 30 min and analyzed by ESI-MS. Significantly, the mass peak for the monosubstituted product, Ru(biq)$_2$(4-azidobutyronitrile)(H_2O), 370.58 was not observed, rather the fully substituted product Ru(biq)$_2$(H_2O)$_2$ was observed even at the shortest light exposures.

Supplementary Figure 11. ESI-MS analysis of Rubpy ligand exchange

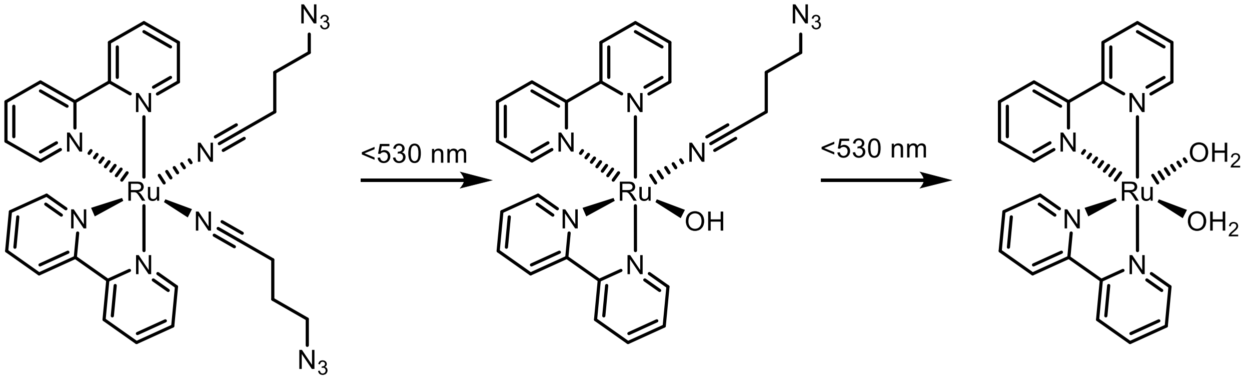

m/2 = 317.08 270.55 225.03

0 min

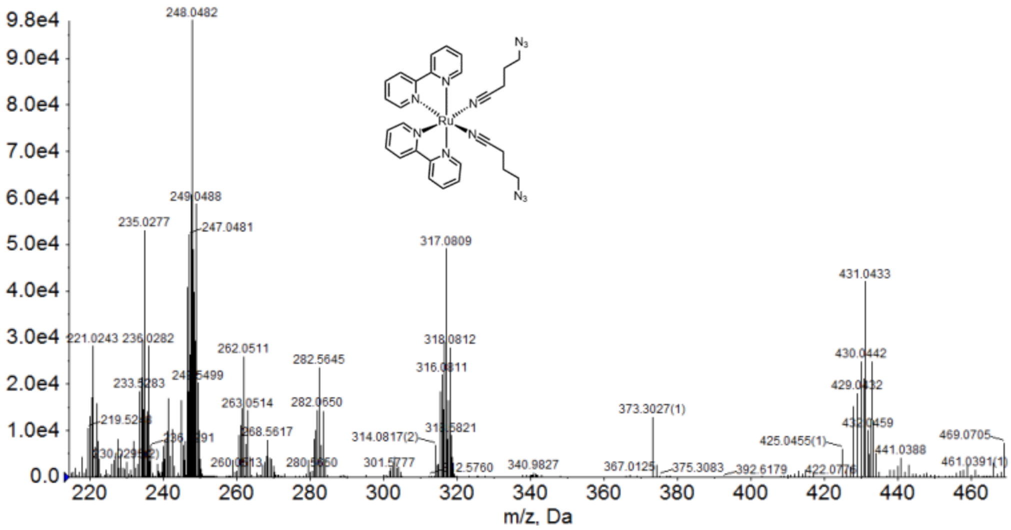

5 min

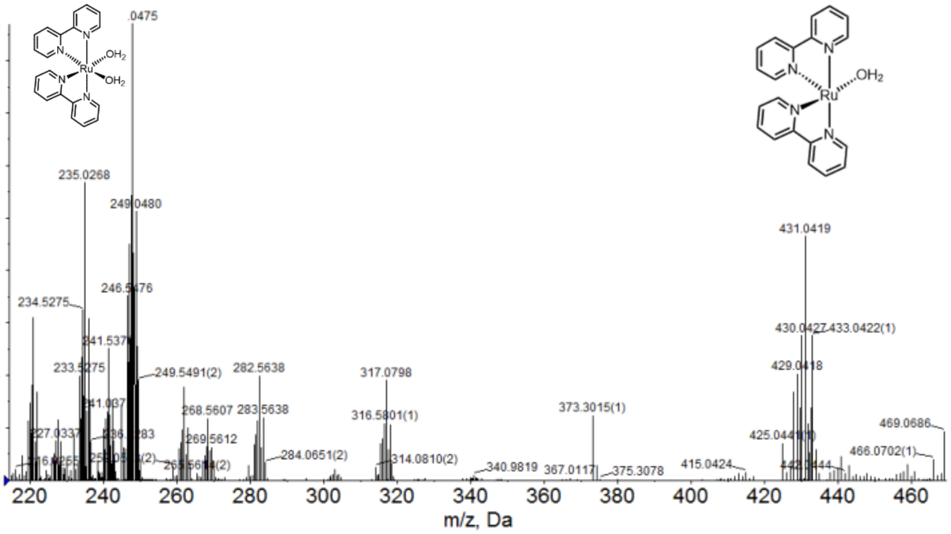

20 min

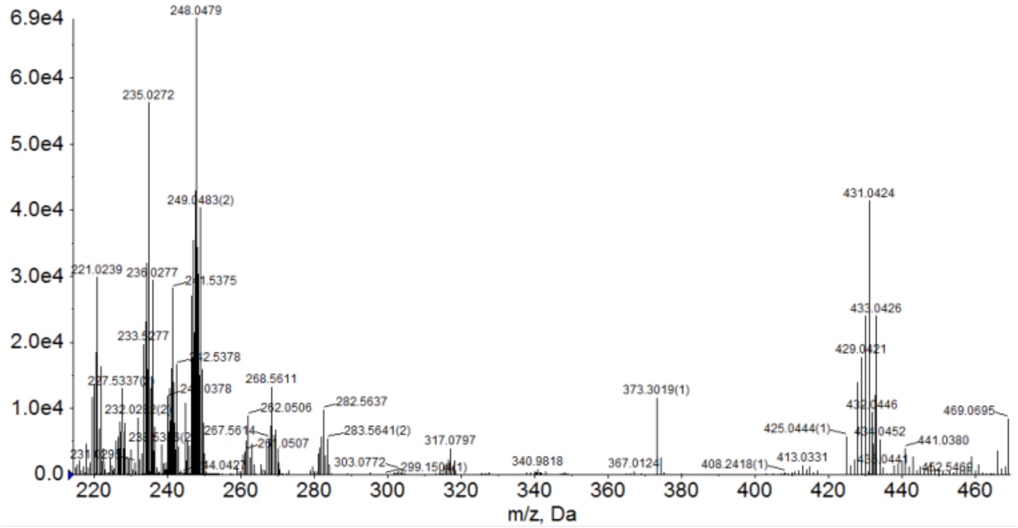

Rubpy (5 mM in H_2O) and exposed to 530 nm light (7.8 mW/cm^2) with constant mixing. Samples were removed at 5 and 20 min and analyzed by ESI-MS. Significantly, the mass peak for the monosubstituted product, $Ru(bpy)_2$(4-azidobutyronitrile)(H_2O), 270.55, was not observed, rather the fully substituted product $Ru(bpy)_2(H_2O)_2$ (225.03) was observed even at the shortest light exposures.

Supplementary Figure 12. Singlet oxygen generation during crosslinker photolysis

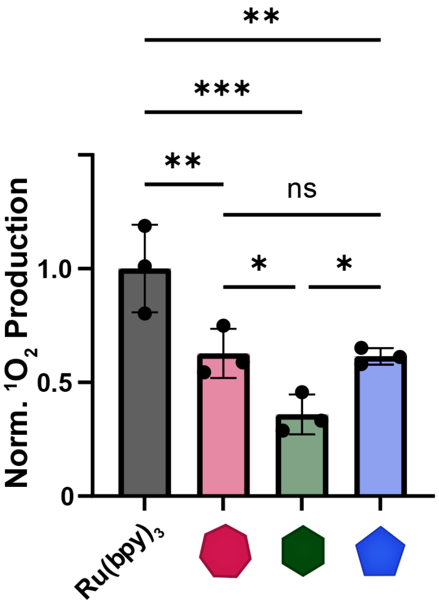

Normalized singlet oxygen production during light exposure for each photocleavable crosslinker reported (Rubiq, pink heptagon; Rubpy, green hexagon; *o*NB, blue pentagon).

Each crosslinker was diluted to a final concentration of 2 µM and mixed with Singlet Oxygen Sensor Green (ThermoFisher) at 0.25 µM. Data was normalized to $^1O_2$ generation by $Ru(bpy)_3$ (2 µM), a known ROS-generating compound. All dye/crosslinker combinations were exposed to 5 mW/cm^2 light of the appropriate wavelength of light (Rubiq 617 nm, Rubpy, 530 nm, *o*NB, 405 nm, $Ru(bpy)_3$, 455 nm) for 30 sec. $^1O_2$ was measured by an increase in Singlet Oxygen Sensor Green fluorescence (ex. 490, em. 530 nm) on a plate reader. (*<0.022, ** <0.0056, ***<0.002, one-way ANOVA) n=3, error bars represent standard deviation about the experimental mean.

Supplementary Figure 13. Hydrogel stability at 37 °C and room temperature

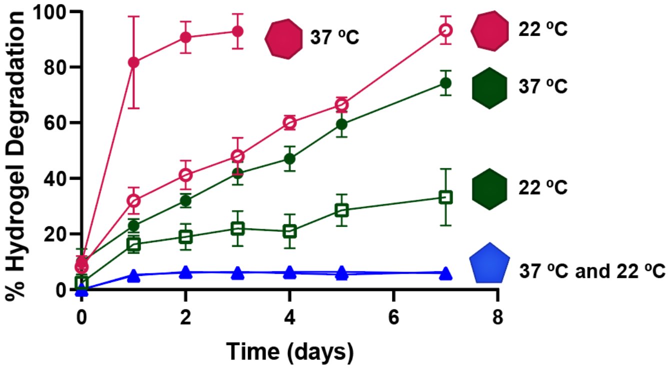

Hydrogel degradation as observed by UV-Vis absorbance at 265 nm (Rubiq), 282 nm (Rubpy), or 216 nm (*o*NB) to detect the presence of crosslinker in the supernatant. 10 µL hydrogels crosslinked with either Rubiq, Rubpy, or *o*NB were submerged in 500 µL sterile PBS and stored in black microcentrifuge tubes at room temperature (22 °C, open shapes) or in a 37 °C incubator (closed shapes) for 7 days. Rubiq hydrogels (pink heptagons) are nearly completely degraded by 24 hr when stored at 37 °C, whereas Rubpy hydrogels (green hexagons) persisted for 5-7 days at body temperature. As expected, *o*NB hydrogels (blue pentagons) are fully stable over the course of 7 days at each temperature. (n=3 hydrogels, error bars represent standard deviation about the experimental mean.)

Supplementary Figure 14. Rheological analysis for TEG-doped hydrogel formation

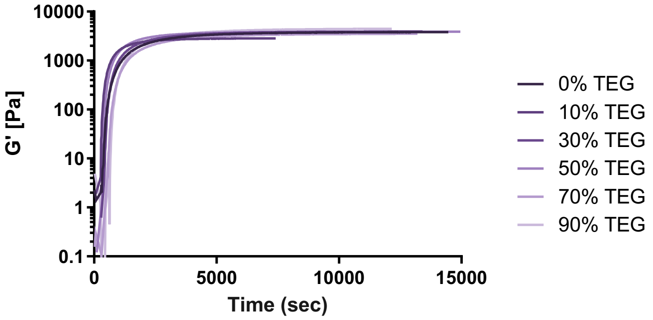

In situ rheometric analysis of hydrogel formation with equal ratios of all three photolabile crosslinkers (i.e., Rubiq, Rubpy, and *o*NB) and increasing amounts of nondegradable diazido-tri(ethylene glycol) (TEG) crosslinker (0 – 90%). Studies performed in the dark.

Supplementary Figure 15. Viability of hS5s following exposure to Rubiq or Rubpy in vitro

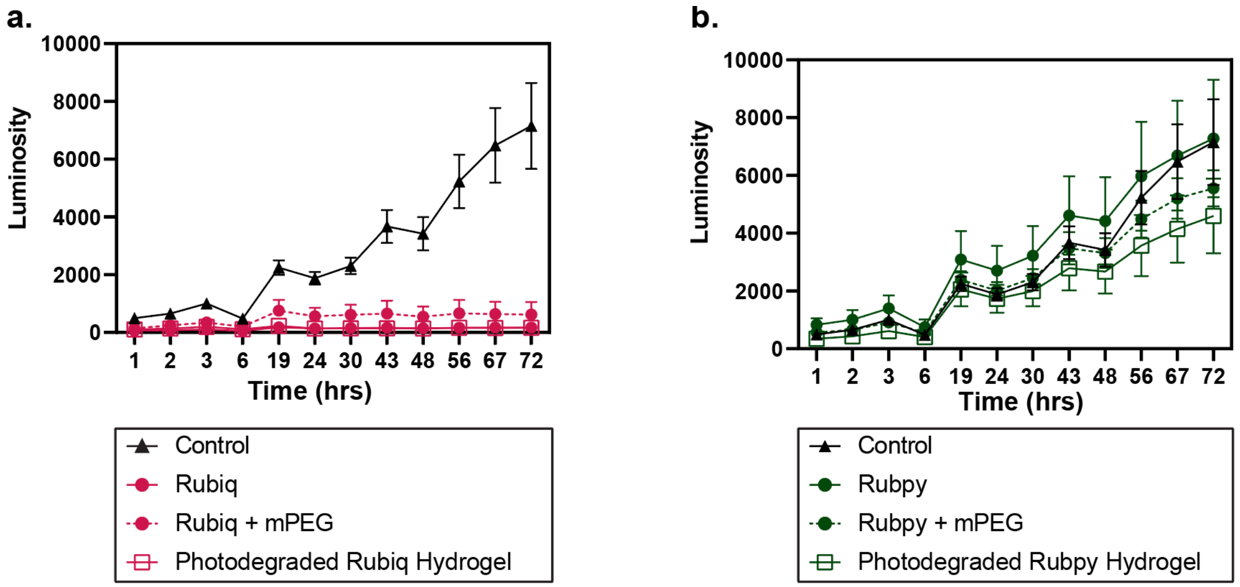

RealTime Glo assay (Promega) was used to assess hS5 cell viability and cell cycle arrest following 3 hr exposure to free Rubiq/Rubpy (solid line, circles). Rubiq/Rubpy individually pre-reacted with 10 kDa monofunctionalized methoxy-PEG-BCN (as previously synthesized[2], 10x, 1 hr, RT, dashed line, circles), or photodegraded hydrogel products (solid line, open squares), all at a final concentration of 1 mM Ru in cell media. **a)** Rubiq showed a significant loss in viability compared to unexposed hS5 cells (control, solid line, triangles), though PEGylation of Rubiq exhibited a protective effect (* p=0.02 between Rubiq and Rubiq-mPEG). The lack of increased viability over time indicates Rubiq's ability to arrest the cell cycle and force cells into a dormant state. Both free Rubiq and Rubiq hydrogel photoproducts were equally toxic to hS5 cells in culture when exposed for over 3 hr. (n=4 biological replicates for each condition, error bars represent standard deviation about the experimental mean.) **b)** While Rubpy hydrogel photoproducts showed some lag in viability, the general increase over time of cell respiration indicates Rubpy does not possess the same ability to arrest cell cycle progression. PEGylation of Rubpy was not found to modulate cellular respiration in a statistically significant manner. (n=4 biological replicates for each condition, error bars represent standard deviation about the experimental mean.)

Supplementary Figure 16. Viability of 10T1/2 fibroblasts following exposure to Rubiq and Rubpy in vitro

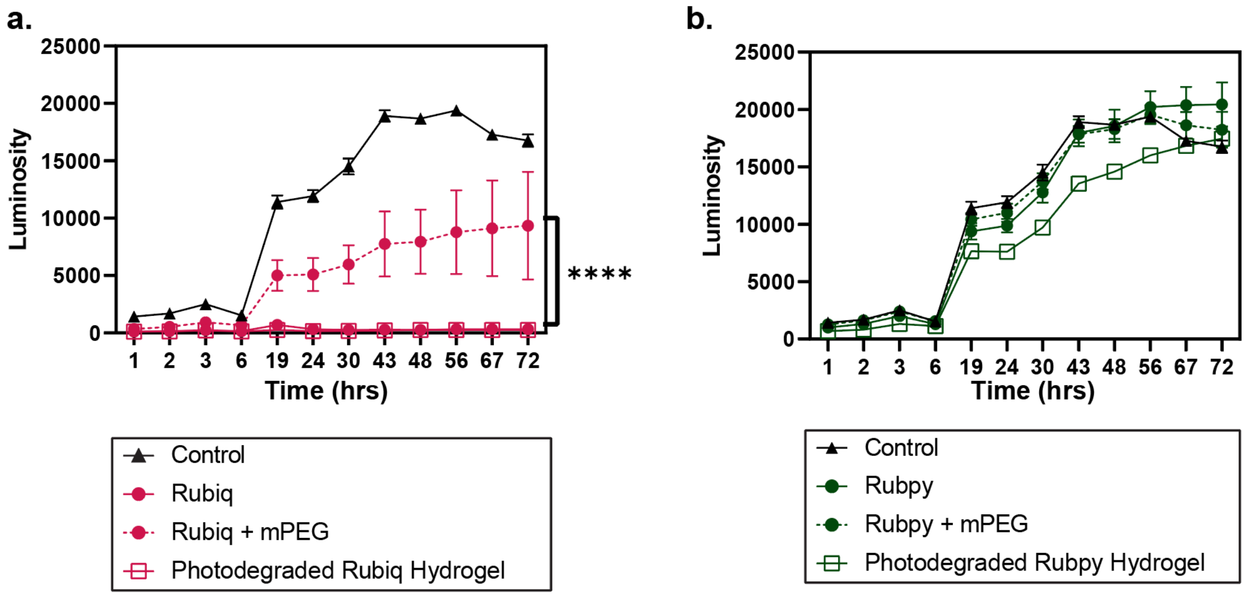

RealTime Glo assay (Promega) was used to assess 10 T1/2 fibroblast cell viability and cell cycle arrest following 3 hr exposure to free Rubiq/Rubpy (solid line, circles), Rubiq/Rubpy individually pre-reacted with 10 kDa monofunctionalized methoxy-PEG-BCN (as previously synthesized[2], 10x, 1 hr, RT, dashed line, circles), or photodegraded hydrogel products (solid line, open squares), all at a final concentration of 1 mM Ru in cell media. **a)** PEGylation of Rubiq shows remarkable protective effect in 10T1/2 fibroblasts, demonstrating significant differences in cellular response between cell types. Both Rubiq and hydrogel photoproducts exhibit significant toxicity at 1 mM levels against 10T1/2 cells. (****<0.0002, one-way ANOVA) (n=4 biological replicates, error bars represent standard deviation about the experimental mean.) **b)** Overall, Rubpy is far less toxic to 10T1/2 cells than Rubiq, with only concentrated hydrogel photoproducts showing a loss in viability over time, which was recovered by the conclusion of this assay. (n=4 biological replicates, error bars represent standard deviation about the experimental mean.)

Supplementary Figure 17. Caspase 3 detection in hS5 cells following exposure to Rubiq or Rubpy in vitro

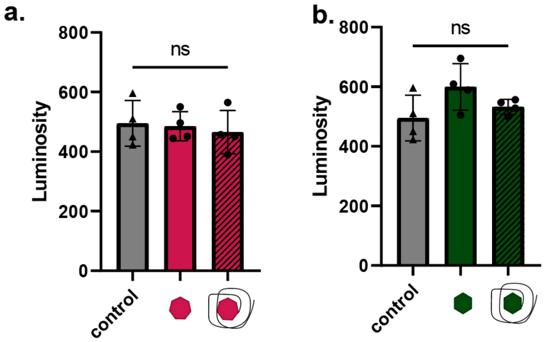

Caspase 3 levels were determined via the ApoTox Glo assay kit (Promega) 24 hours after exposure to Rubiq (shown in part **a**) or Rubpy (shown in part **b**) (1 mM, 3 hr exposure, solid bars), or Rubiq/Rubpy prereacted with a monofunctionalized methoxy-PEG-BCN (1 mM Ru concentration, 3 hr exposure, diagonal line bar). Compared to control cells (grey, triangles) caspase levels were not significantly different ($p > 0.05$, one-way ANOVA), suggesting that cell death proceeds via necrosis or catastrophic loss of membrane integrity rather than organized apoptosis. (n=4 biological replicates, error bars represent standard deviation.)

Supplementary Figure 18. Caspase 3 detection in 10T1/2 cells following exposure to Rubiq or Rubpy in vitro

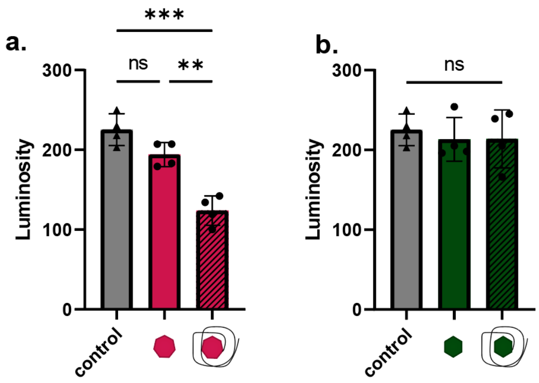

Caspase 3 levels were determined via the ApoTox Glo assay kit (Promega) 24 hours after exposure to Rubiq (shown in part **a**) or Rubpy (shown in part **b**) (1 mM, 3 hr exposure, solid bars), or Rubiq/Rubpy prereacted with monofunctionalized methoxy-PEG-BCN (1 mM Ru concentration, 3 hr exposure, diagonal line bar). Compared to control cells (grey, triangles) caspase levels for Rubiq and Rubpy were not significantly different ($p > 0.05$, one-way ANOVA), suggesting that cell death proceeds via necrosis or catastrophic loss of membrane integrity rather than organized apoptosis. Interestingly, a significant difference (** p=0.0002, *** p<0.0001, two-tailed Welch's t test) was observed for the PEGylated Rubiq relative to the free Rubiq and control. (n=4 biological replicates, error bars represent standard deviation about the experimental mean.)

Supplementary Figure 19. Viability and proliferation of hMSCs following hydrogel encapsulation and release

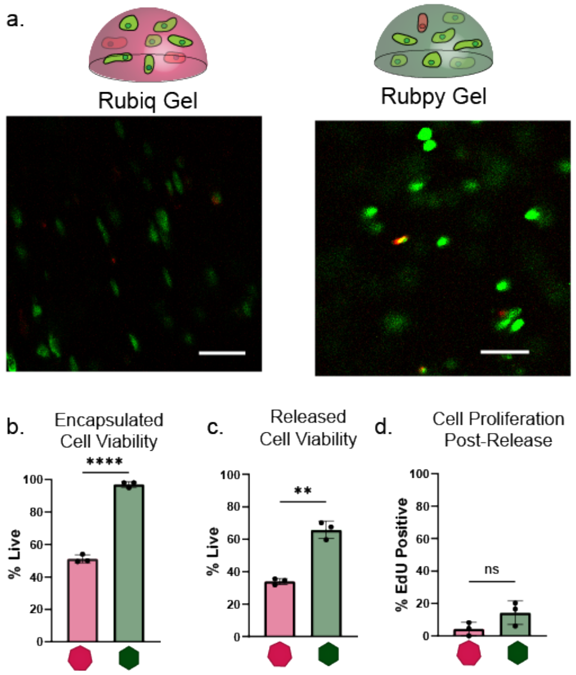

Live/dead statistics as determined by ethidium homodimer/calcein green staining. **a)** Images of human mesenchymal stem cells (hMSCs, passage 7-11) in Rubiq- and Rubpy-crosslinked PEG hydrogels respectively. (Scale bar = 100 μm) **b)** Cell count data extracted from cell-laden hydrogels stained for live and dead cells. (**** $p<0.0001$, two-tailed Welch's t test) **c)** hMSCs collected from hydrogels degraded with 617 nm and 530 nm light respectively (10 mW/cm^2, 1-3 min), subsequently stained for live/dead and analysed by flow cytometry. (** $p=0.0002$, two-tailed Welch's t test) **d)** Cell proliferation observed by EdU staining tagging new DNA, performed on cells released from hydrogels. (≥100 nuclei counted) ($p>0.05$, two-tailed Welch's t test) ($n=3$ biological replicates, error bars represent standard deviation about the experimental mean.)

Supplementary Figure 20. Viability and proliferation of hS5 cells following hydrogel encapsulation and release

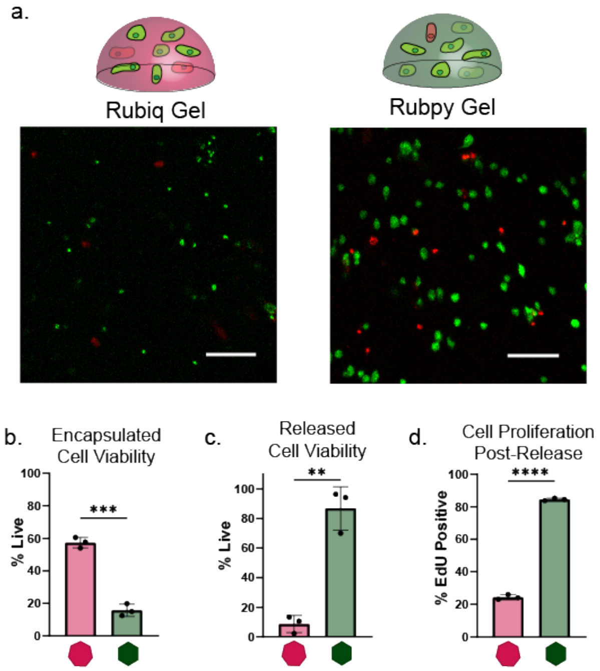

Live/dead statistics as determined by ethidium homodimer/calcein green staining. **a)** Images of human stromal cells (hS5, passage 4-5) in Rubiq- and Rubpy-crosslinked PEG hydrogels respectively. (Scale bar = 100 μm) **b)** Cell count data extracted from cell-laden hydrogels stained for live and dead cells. (*** $p<0.0001$, two-tailed Welch's t test) **c)** hMSCs collected from hydrogels degraded with 617 nm and 530 nm light respectively (10 mW/cm^2, 1-3 min), subsequently stained for live/dead and analysed by flow cytometry. (** $p=0.0002$, two-tailed Welch's t test) **d.** Cell proliferation observed by EdU staining tagging new DNA, performed on cells released from hydrogels. (≥100 nuclei counted) (**** $p<0.0001$, two-tailed Welch's t test) (n=3 biological replicates, error bars represent standard deviation about the experimental mean.)

Supplementary Figure 21. Gating strategy for measuring live/dead cell populations via flow cytometry

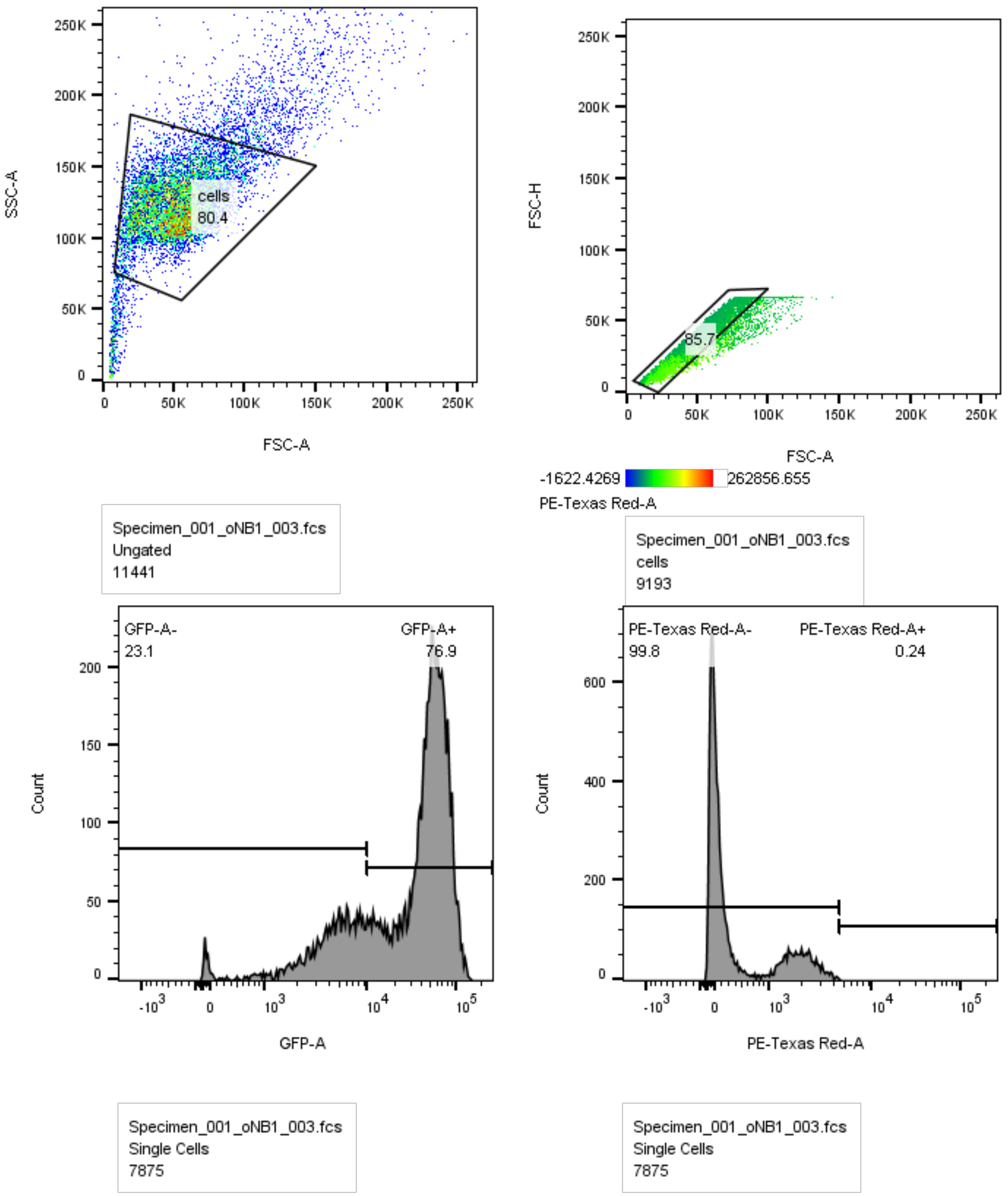

Gating strategy to determine percent live/dead for cell populations released from *o*NB, Rubpy, and Rubiq hydrogels (*o*NB hydrogel data shown here).

Supplementary Figure 22. Gating strategy for cell population quantification via flow cytometry

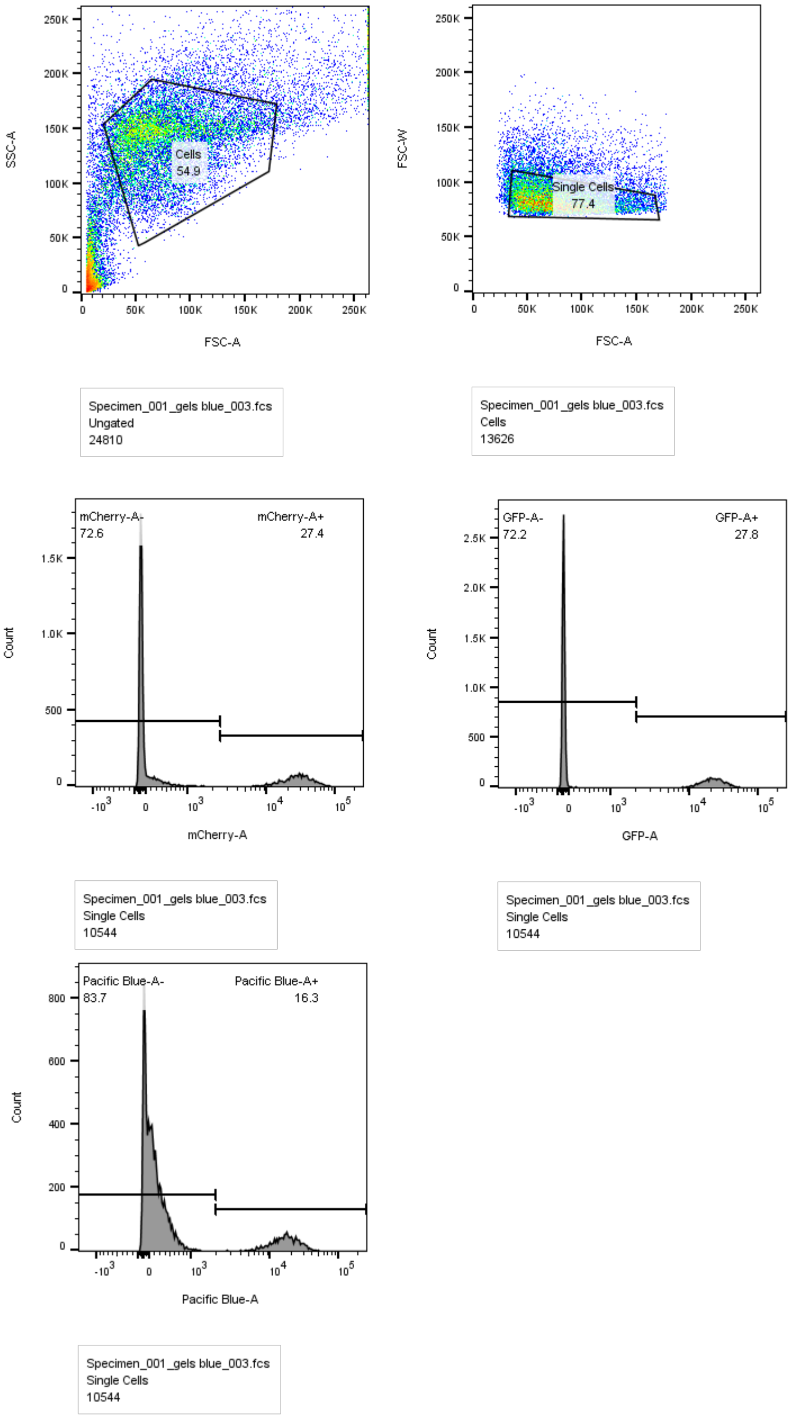

Gating strategy to identify mCherry^{+}, GFP^{+}, and BFP^{+} cells released from hydrogels under different wavelengths of light exposure. Blue (405 nm) light exposure shown here.

Supplementary references

1. Badeau, B. A., Comerford, M. P., Arakawa, C. K., Shadish, J. A. & DeForest, C. A. Engineered modular biomaterial logic gates for environmentally triggered therapeutic delivery. *Nature Chemistry* **10,** 251–258 (2018).
2. Shadish, J. A., Benuska, G. M. & DeForest, C. A. Bioactive site-specifically modified proteins for 4D patterning of gel biomaterials. *Nature Materials* **18,** 1005–1014 (2019).
