# Supplementary material for: Tricolor visible wavelength-selective photodegradable hydrogel biomaterials
Source: Nat Commun. 2023 Aug 29;14:5250. doi: 10.1038/s41467-023-40805-w (PMC10462736; doi:10.1038/s41467-023-40805-w)
Supplement: Supplementary file 2 — Reporting Summary [file 41467_2023_40805_MOESM2_ESM.pdf]

nature portfolio

Corresponding author(s): Cole A. DeForest

Last updated by author(s): Aug 1, 2023

Reporting Summary

Nature Portfolio wishes to improve the reproducibility of the work that we publish. This form provides structure for consistency and transparency in reporting. For further information on Nature Portfolio policies, see our Editorial Policies and the Editorial Policy Checklist.

Statistics

For all statistical analyses, confirm that the following items are present in the figure legend, table legend, main text, or Methods section.

n/a	Confirmed	
☐	☒	The exact sample size (*n*) for each experimental group/condition, given as a discrete number and unit of measurement
☐	☒	A statement on whether measurements were taken from distinct samples or whether the same sample was measured repeatedly
☐	☒	The statistical test(s) used AND whether they are one- or two-sided<br>*Only common tests should be described solely by name; describe more complex techniques in the Methods section.*
☒	☐	A description of all covariates tested
☒	☐	A description of any assumptions or corrections, such as tests of normality and adjustment for multiple comparisons
☐	☒	A full description of the statistical parameters including central tendency (e.g. means) or other basic estimates (e.g. regression coefficient) AND variation (e.g. standard deviation) or associated estimates of uncertainty (e.g. confidence intervals)
☐	☒	For null hypothesis testing, the test statistic (e.g. *F*, *t*, *r*) with confidence intervals, effect sizes, degrees of freedom and *P* value noted<br>*Give P values as exact values whenever suitable.*
☒	☐	For Bayesian analysis, information on the choice of priors and Markov chain Monte Carlo settings
☒	☐	For hierarchical and complex designs, identification of the appropriate level for tests and full reporting of outcomes
☒	☐	Estimates of effect sizes (e.g. Cohen's *d*, Pearson's *r*), indicating how they were calculated

Our web collection on statistics for biologists contains articles on many of the points above.

Software and code

Policy information about availability of computer code

Data collection	Data collection was performed with the assistance of NanoDrop2000/2000c 1.6.198, Bruker Topspin 2.1, Gen5 Ver 3.09, Azure Biosystems 1.6.4.1229, DataMax V2.2, Leica Application Suite X 4.5.0.25531, and Analyst TF 1.7.1.
Data analysis	Data analysis was performed with the assistance of Microsoft Excel 2016, GraphPad Prism Version 6, Bruker Topspin 2.1, ImageJ 1.53f51, Adobe Illustrator CS6 Version 16.0.0, CellProfiler 4.2.1, ChemDraw 20.0, and FlowJo 10.

For manuscripts utilizing custom algorithms or software that are central to the research but not yet described in published literature, software must be made available to editors and reviewers. We strongly encourage code deposition in a community repository (e.g. GitHub). See the Nature Portfolio guidelines for submitting code & software for further information.

Data

Policy information about availability of data

All manuscripts must include a data availability statement. This statement should provide the following information, where applicable:

- Accession codes, unique identifiers, or web links for publicly available datasets
- A description of any restrictions on data availability
- For clinical datasets or third party data, please ensure that the statement adheres to our policy

All pertinent experimental procedures, materials, methods, and characterization data are available within this manuscript and its associated Supplementary Information. Source data are provided with this paper.

Human research participants

Policy information about studies involving human research participants and Sex and Gender in Research.

Reporting on sex and gender	n/a
Population characteristics	n/a
Recruitment	n/a
Ethics oversight	n/a

Note that full information on the approval of the study protocol must also be provided in the manuscript.

Field-specific reporting

Please select the one below that is the best fit for your research. If you are not sure, read the appropriate sections before making your selection.

☒ Life sciences ☐ Behavioural & social sciences ☐ Ecological, evolutionary & environmental sciences

For a reference copy of the document with all sections, see nature.com/documents/nr-reporting-summary-flat.pdf

Life sciences study design

All studies must disclose on these points even when the disclosure is negative.

Sample size	No statistical method was used to predetermine sample size. All experiments were performed at a minimum in biological triplicate.
Data exclusions	No data was excluded.
Replication	All attempts at replication were successful. All experiments were performed at a minimum in biological triplicate.
Randomization	No randomizations were required for the experiments performed. A positive and negative control were used when necessary.
Blinding	Blinding was not relevant to the study as there were no relevant biases. There were no participants in any study so blinding was not necessary for these studies.

Reporting for specific materials, systems and methods

We require information from authors about some types of materials, experimental systems and methods used in many studies. Here, indicate whether each material, system or method listed is relevant to your study. If you are not sure if a list item applies to your research, read the appropriate section before selecting a response.

Materials & experimental systems

n/a	Involved in the study	
☒	☐	Antibodies
☐	☒	Eukaryotic cell lines
☒	☐	Palaeontology and archaeology
☒	☐	Animals and other organisms
☒	☐	Clinical data
☒	☐	Dual use research of concern

Methods

n/a	Involved in the study	
☒	☐	ChIP-seq
☐	☒	Flow cytometry
☒	☐	MRI-based neuroimaging

Eukaryotic cell lines

Policy information about cell lines and Sex and Gender in Research

Cell line source(s)	10T1/2 fibroblast-like cells isolated from mouse embryo cells were gifted by Dr. Jennifer Davis (University of Washington). Fluorescent and non-fluorescent hS5 human stromal cells (from a White, 30-year-old, male) were gifted by Dr. Beverly Torok-Starb (Fred Hutch) (originally purchased from ATCC). Human bone-marrow derived mesenchymal stem cells (hMSCs) were purchased from Sigma Aldrich.

Authentication	Cell line was authenticated by supplier (morphology, STR profiling).
Mycoplasma contamination	Cell line used in these studies were not tested for mycoplasma contamination.
Commonly misidentified lines (See ICLAC register)	No commonly misidentified cell lines were used in the study

Flow Cytometry

Plots

Confirm that:

☒ The axis labels state the marker and fluorochrome used (e.g. CD4-FITC).

☒ The axis scales are clearly visible. Include numbers along axes only for bottom left plot of group (a 'group' is an analysis of identical markers).

☒ All plots are contour plots with outliers or pseudocolor plots.

☒ A numerical value for number of cells or percentage (with statistics) is provided.

Methodology

Sample preparation	Cells were released into solution via trypsin digest or gel photodegradation, washed with PBS and resuspended in PBS for analysis.
Instrument	BD LSRII
Software	FlowJo v10
Cell population abundance	Flow cytometry was used to quantify fluorescent cell release from photodegradable hydrogels. All recovered cells were included in analysis.
Gating strategy	Live cells were gates between FSC-A 10k-150k, SSC-A 50k-100k, single cells selected between FSC-A 50k-150k, FSC-W 50k-100k. Gates to determine positive and negative presentation were set on histograms near 10^4.

☒ Tick this box to confirm that a figure exemplifying the gating strategy is provided in the Supplementary Information.
